# Supplementary material for: A reversibly gated protein-transporting membrane channel made of DNA
Source: Nat Commun. 2022 Apr 28;13:2271. doi: 10.1038/s41467-022-28522-2 (PMC9051096; doi:10.1038/s41467-022-28522-2)
Supplement: Supplementary file 1 — Supplementary Information [file 41467_2022_28522_MOESM1_ESM.pdf]

A Reversibly Gated Protein-Transporting Membrane Channel Made of DNA

Swarup Dey[1,2,3#], Adam Dorey[4#], Leeza Abraham[1,2#], Yongzheng Xing[4], Irene Zhang[1], Fei Zhang[5], Stefan Howorka[4]*, Hao Yan[1,2]*

[1]Biodesign Center for Molecular Design and Biomimetics (at the Biodesign Institute) at Arizona State University, Tempe, AZ 85287, USA.

[2]School of Molecular Sciences, Arizona State University, Tempe, AZ 85287, USA.

[3] Present address: Wyss Institute for Biologically Inspired Engineering, Harvard University, Boston, MA 02115, USA.

[4]Department of Chemistry & Institute of Structural Molecular Biology, University College London, UK

[5]Department of Chemistry, Rutgers University, Newark, NJ 07102, USA.

[#]These authors contributed equally: Swarup Dey, Adam Dorey, Leeza Abraham

These authors jointly supervised this work: Stefan Howorka, Hao Yan

*email: s.howorka@ucl.ac.uk, hao.yan@asu.edu

Supplementary Information

Contents

Design specifications of Large Diameter Nanopore ***5***

Positions of cholesterol modification on the nanopore ***7***

Flexible hinge design to facilitate dynamic opening and closing of the LGC lid ***9***

Toehold-mediated strand displacement scheme used for reversible opening and closing of LGC lid. ***10***

Agarose gel electrophoresis characterization of nanopore formation ***12***

AFM characterization and oxDNA simulation of LGC formation ***13***

TEM characterization of LGC formation ***14***

Supplementary Note 1: Agarose gel electrophoresis analysis of the interaction of LGC-N pore with SUVs under different conditions ***15***

AFM characterization of LGC in no Mg^{2+} buffer ***17***

Interaction of LGC with lipid membrane: large field of view TEM images ***18***

Confocal images showing proper focus during course of imaging for GUV-atto 633 dye influx assay through Cy3-labelled LGC in main text Fig. 2c-ii ***19***

FRET assay to demonstrate membrane spanning and stable channel formation by cholesterol modified LGC ***20***

Single traces showing kinetics of atto-633 dye/GFP influx into GUVs ***22***

Supplementary note 2: Kinetics of dye influx ***23***

No cap DNA plate as control to show that the cholesterol modifications are not disturbing the membrane or making it leaky *25*

Negative control no-cap DNA plate *26*

Conductance states of LGC-N *27*

Cyclic opening and closing of the lidded LGC Negative control with lid opening using mismatch opening key *28*

Supplementary note 3: Kinetics of lid opening by FRET *29*

Negative controls for atto-633 dye influx experiments in main text Fig. 3b-ii: LGC with closed and opened lid without cholesterol modification *34*

Negative control influx assays using mismatch key *35*

Two dye influx to show dynamic closing of the lid *36*

All-point histogram analysis for single channel current recordings of open lid pore *37*

Noise comparison of LGC-C and LGC-O *38*

Noise comparison of LGC-O at positive and negative potentials *39*

Electrophysiological characterization of lid opening-closing *40*

Electrophysiological characterization of lid opening-closing *41*

GFP influx through LGC with no lid (LGC-N) *42*

Negative controls for GFP influx experiments in main text Fig. 4a-ii: LGC with no lid, closed lid and opened lid without cholesterol modification *43*

Negative control to show size dependent transport *44*

Cargo release through LGC* *45

Rate of influx%* *46

Analysis of the interaction of trypsin with LGC-C* *47

Increasing trypsin concentration leads to increased translocation events per second* *48

Analysis of the voltage-dependent translocation of trypsin through LGC-N* *49

pH dependence of GFP translocation through LGC-N* *50

Analysis of GFP translocation through LGC-N at pH 4.5* *51

Size comparison of the ions and dye used to the oxDNA simulated LGC-C* *52

Supplementary References* *53

Design specifications of Large Diameter Nanopore

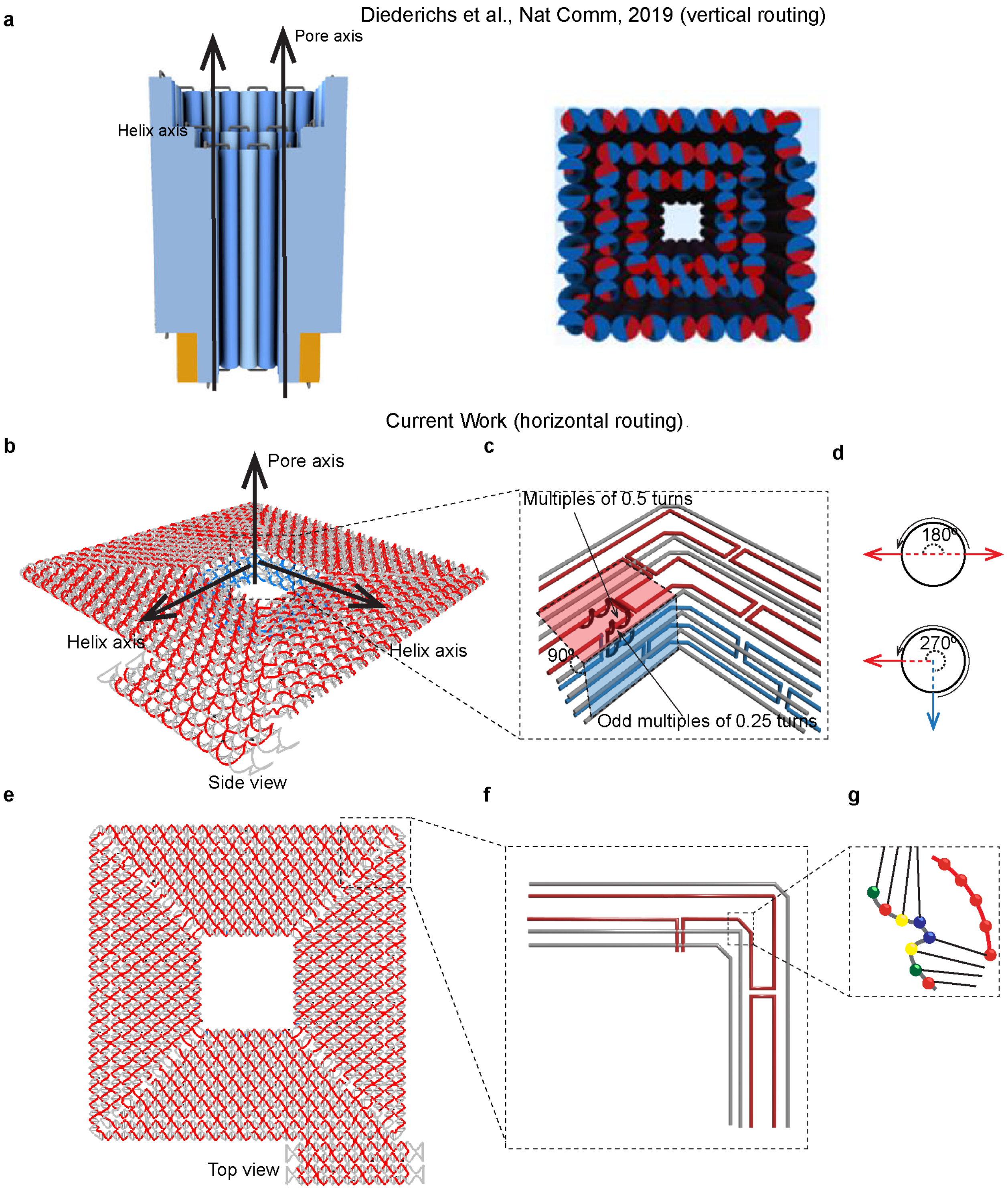

Supplementary Figure 1: Design novelty of Large Diameter Nanopore. (**a**) Side view (left) and top view (right) of the nanopore of Diederichs *et al*, Nat Comm, 2019,[1] exemplifying routing of helices in previously published DNA nanopores. The helical axis of the component helices ran vertically in these DNA nanopores, i.e. – parallel to the vertical axis of the nanopore. (**b**) Side view of LGC. Grey, red and blue strands represent, respectively - M13 scaffold, plate forming staples and pore forming staples. The pore

forming helices run horizontally, i.e. – perpendicular to the pore axis. This horizontal routing ensures fine tuning of the pore diameter as well as having a large surface area of the plate, available for cholesterol placement. (**c**) Zoomed-in image of the pore region showing layered crossover design to form the plate and the pore of LGC, routed in mutually normal planes (red plane – plate forming helices, blue plane – pore forming helices). The distance between two interhelical crossovers determines their relative spatial alignment. Hence, we create interhelical crossovers spaced apart in a multiple of 0.5 turns, i.e. - 1 × 0.5 turns = 6nt; 2 × 0.5 turns = 10.5nt; 3 × 0.5 turns = 16nt etc. for the helices on the same plane (i.e – among plate forming helices in the red plane and among pore forming helices in the blue plane). Whereas the gap between a crossover in the red 'plate plane' and a crossover in the blue 'pore plane' is kept as an odd multiple of 0.25 turns, except 1 × 0.25 turns i.e. – 3 × 0.25 turns = 8nt; 5 × 0.25 turns = 13nt; 7 × 0.25 turns = 18nt etc. This ensures the red 'plate plane' and the blue 'pore plane' to be perpendicular to each other. (**d**) The direction of the helices at crossover points. Circle represents top view of a helix and arrows represent tangent drawn to a helix at a given point to show its direction, red arrow – helices in plate plane and blue arrows – helices in pore plane. Top panel – relative angle between the helical direction at two crossover points in the plate plane. Bottom panel – relative angle between the direction of a helix at a crossover in the plate plane and a crossover in the pore plane. (**e**) Top view of the pore. (**f**) Zoomed-in view of the corners where a 4 base loops in only one of the two strands in each helix imposes a 90º curvature in the same plane. (**g**) Zoom in view of a corner crossover showing the 4 base loop insertion strategy.

Positions of cholesterol modification on the nanopore

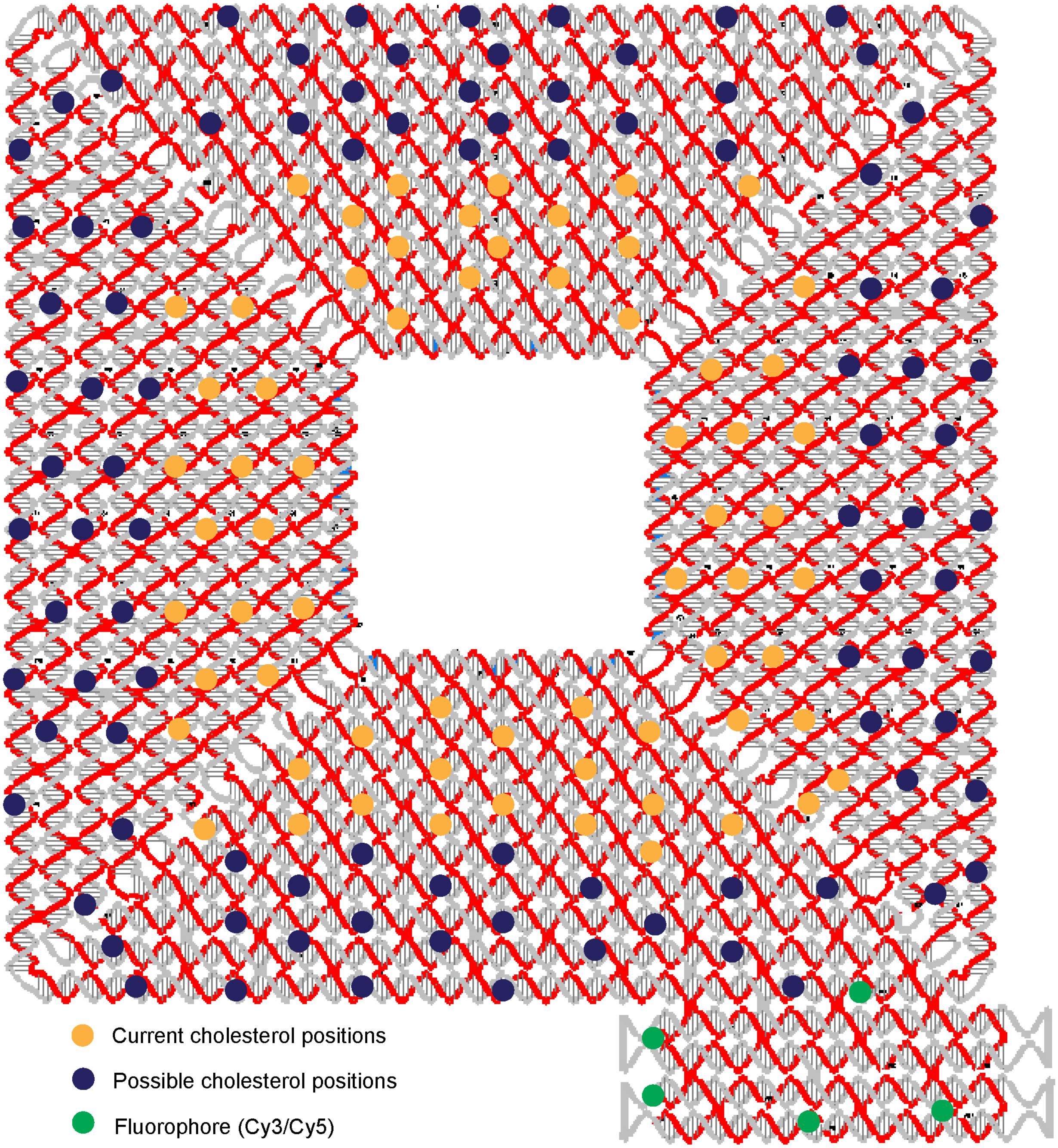

Supplementary Figure 2: Position of hydrophobic cholesterol anchors (orange spheres) and optional fluorophore modification (green spheres) in the large diameter nanopore. According to Thompson *et al,* Nat Comm, 2019's continuum model and MD simulation predictions[2] (Supplementary Fig. 2, Thomson et al, Nat Comm 2019) a pore of ~20nm width would require > 60 cholesterol probes. Thus, we used 64 cholesterol probes. Handles of specific sequence is extended from the 64 denoted locations, ensuring the downward orientation of the helices at the location. When cholesterol bearing anti-handle strand is added, their binding at the handle strand bearing locations tag the nanopore with 64 cholesterol molecules that help its insertion into the lipid bilayer. Although, this work only uses the denoted locations, the horizontally routed flat design offers more place that can

accommodate at least 96 more cholesterol molecules (blue spheres) for future design of larger pore. The fluorophores are placed in the index region to ensure sufficient distance from the functional regions of the nanopore to avoid any undesired non-specific interactions.

Flexible hinge design to facilitate dynamic opening and closing of the LGC lid

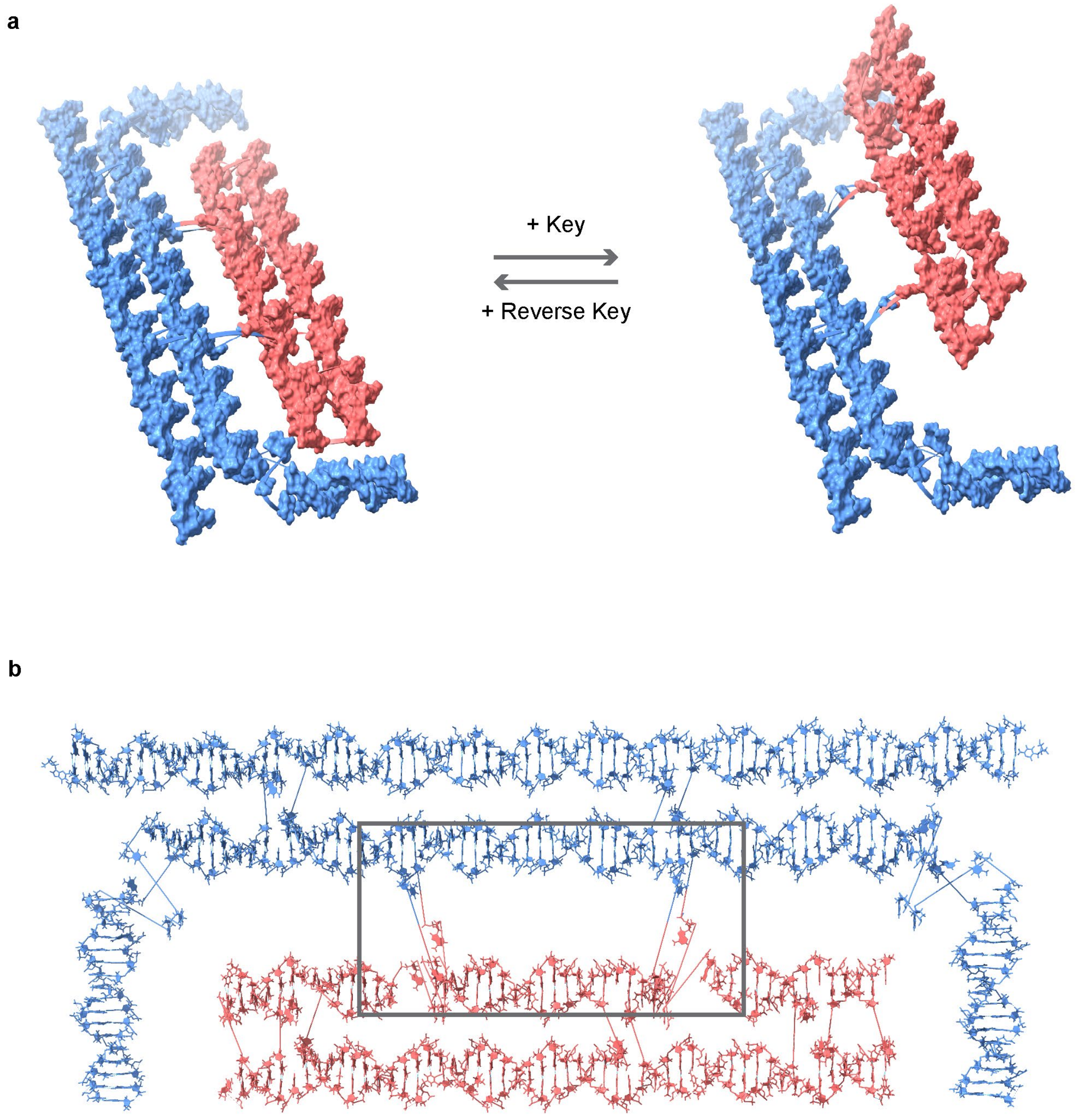

Supplementary Figure 3: (**a**) Side view and (**b**) top view of the optimally designed flexible hinge of the LGC lid formed by a 4-nucleotide single stranded region in each hinge. Several designs with rigid hinge (data not shown) did not lead to successful closure of the lid, perhaps because of strain in the hinge region.

Toehold-mediated strand displacement scheme used for reversible opening and closing of LGC lid.

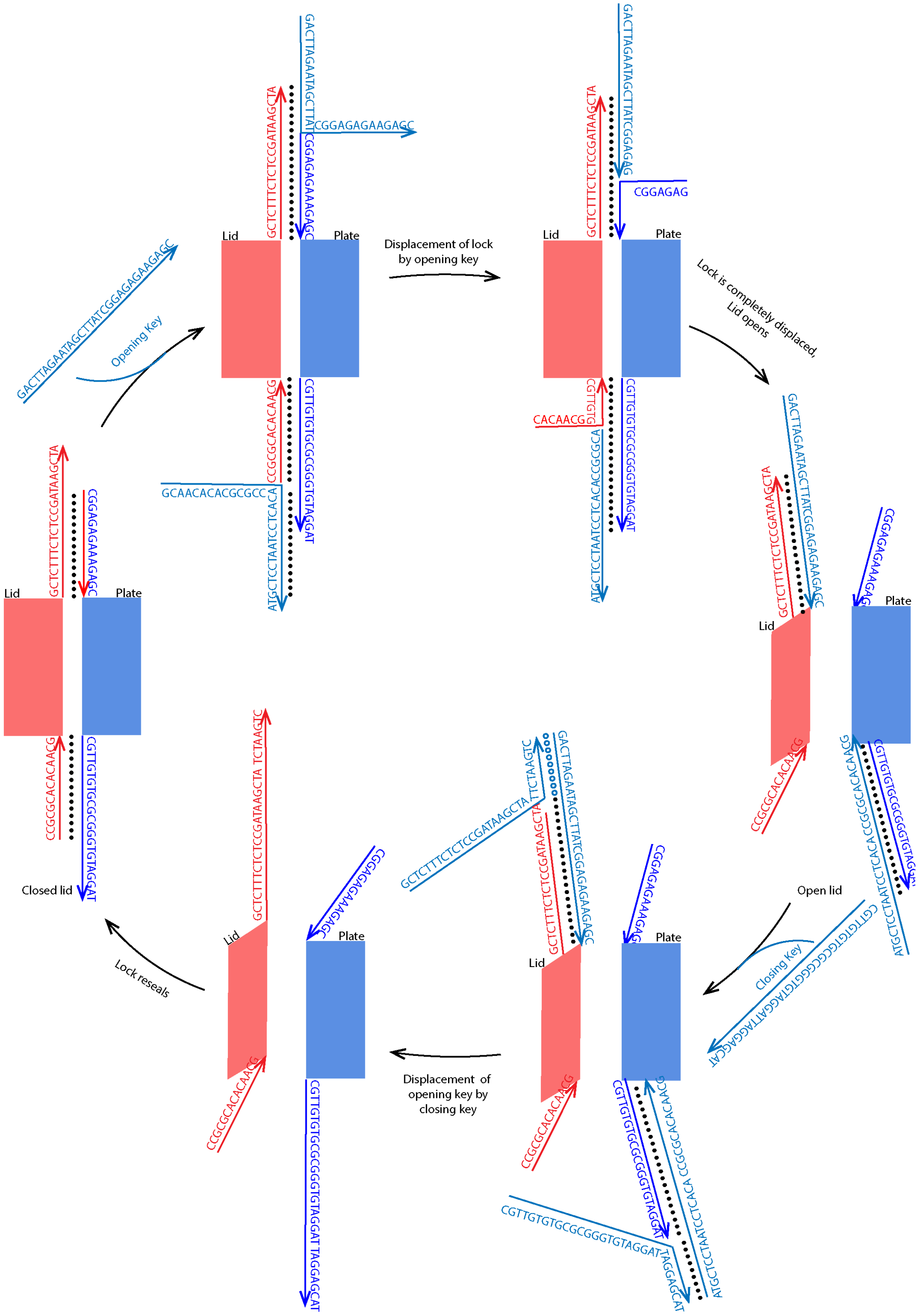

Supplementary Figure 4: Details of the strand displacement reaction for reversible lid opening and closing. Lid (pink) and plate (blue) are locked in two positions, each lock containing a strand with a toehold. Opening key (light blue) opens the lock by toehold mediated strand displacement (TMSD), leading to open lid. The opening key also contains a toehold which then initiates a second TMSD when closing keys (light blue) are added. As a result, the opening key dissociates from the lock strands and lock strands reseal to form the closed lid.

Agarose gel electrophoresis characterization of nanopore formation

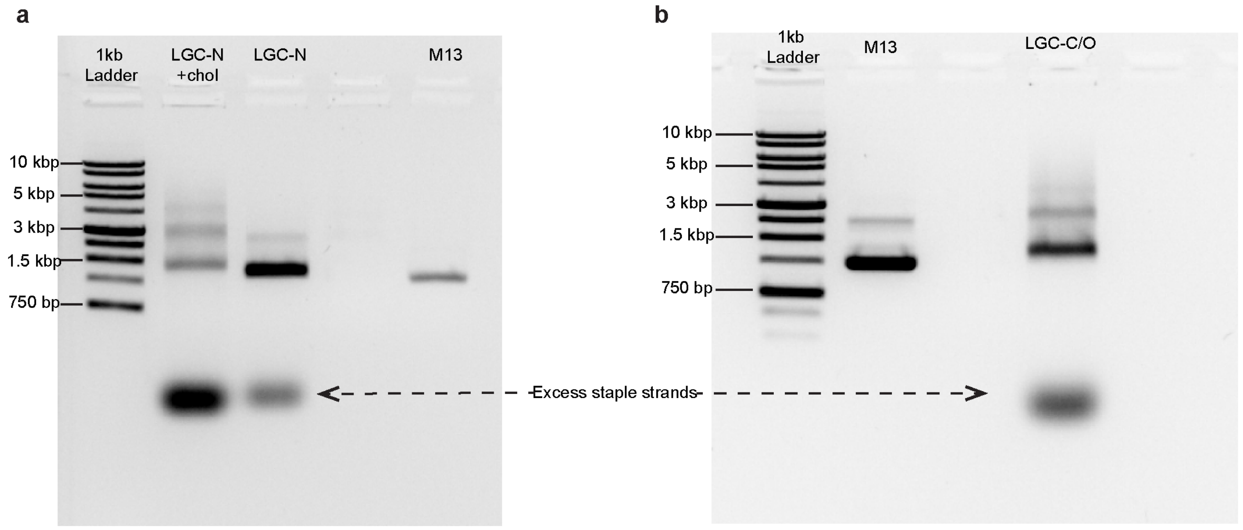

Supplementary Figure 5: 1.5% Agarose gel electrophoresis characterization of the formation of the large diameter nanopores (LGC). (**a**) No lid-LGC with cholesterol (LGC-N +chol) and without cholesterol (LGC-N). Slight upshift of the LGC-N with respect to the scaffold M13 shows its correct formation with a predominant monomer band a slight dimer band. The successful cholesterol modification is shown by upshift of monomer as well as dimer bands of LGC-N +Chol compared to the LGC-N band and increased smearing due to aggregation in case of LGC-N +Chol. (**b**) Formation of non-cholesterol LGC with lid (LGC-C/O) in 1.5% agarose gel. The predominant band of LGC-C/O with lid upshifts compared to that for LGC-N which indicates successful formation of the lid. The experiments were repeated more than 10 times achieving the same result. Source data are provided as a Source Data file.

AFM characterization and oxDNA simulation of LGC formation

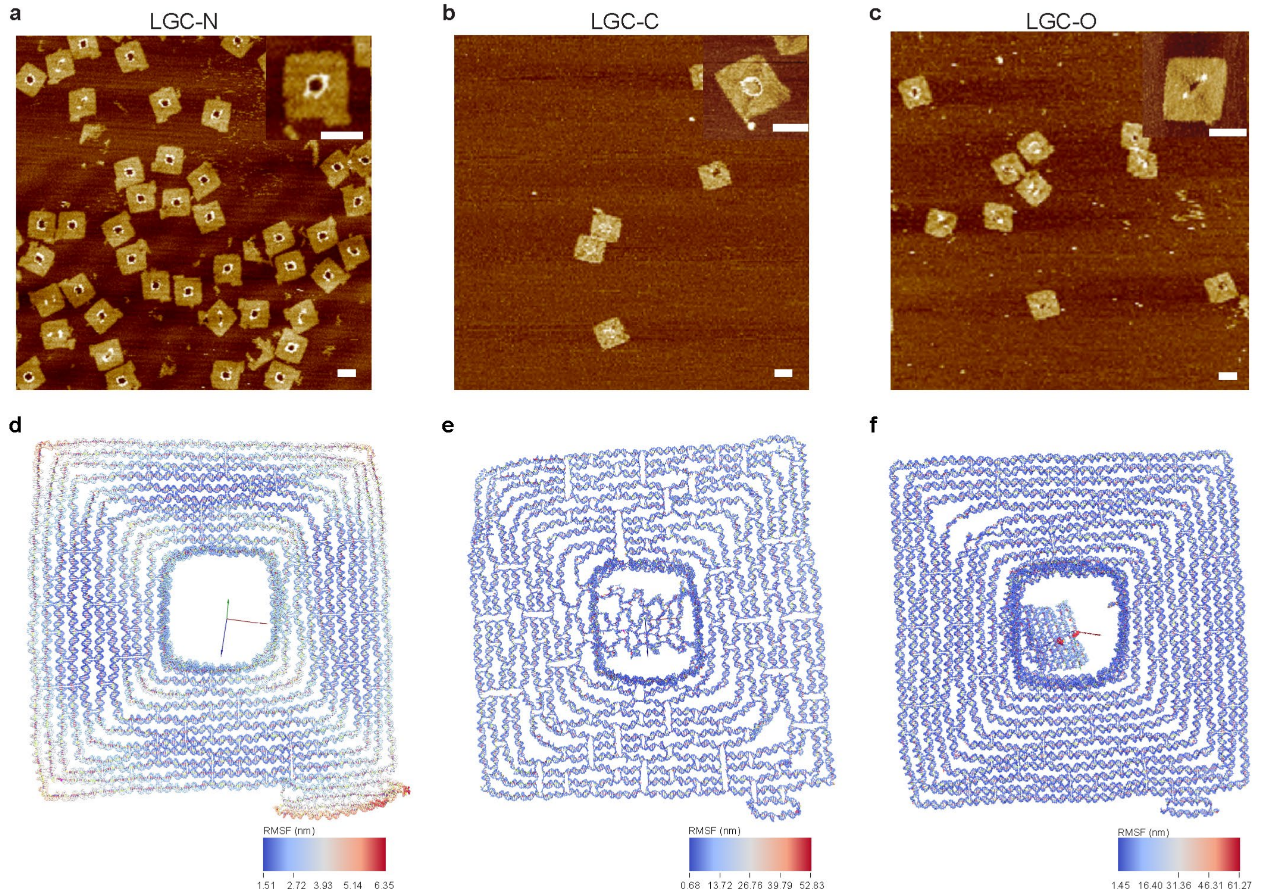

Supplementary Figure 6: (a-c) AFM images and (**d-f**) mean structure and root mean squared fluctuations (RMSF) obtained from oxDNA simulations for non-cholesterol versions of – (**a,d**) LGC without lid (LGC-N) (**b,e**) LGC with closed lid (LGC-C) and (**c,f**) LGC with open lid (LGC-O). AFM scale bars: 50 nm. The data in (**a-c**) is representative of n=2 independent experiments each.

TEM characterization of LGC formation

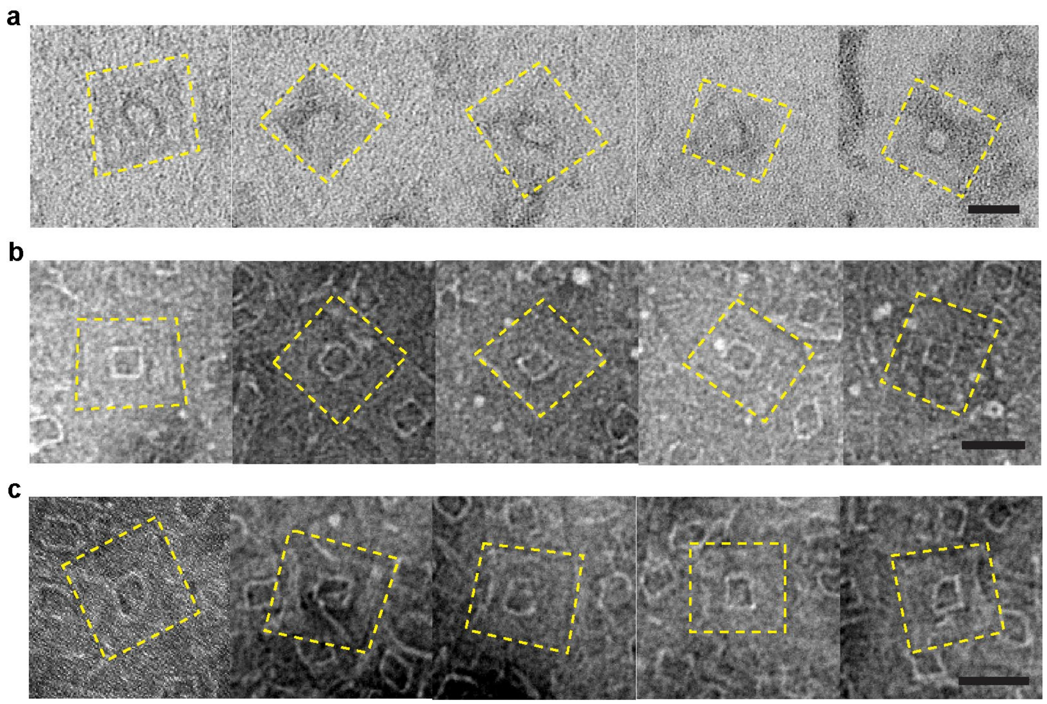

Supplementary Figure 7: TEM characterization of non-cholesterol LGC. (**a**) Without lid. (**b**) With closed lid and (**c**) With open lid. The edge of the nanopore is annotated with dashed line for ease of understanding. The pore in the middle of the square is visible in all cases. The pore is empty in case of LGC without lid (**a**). A lid is clearly visible in the middle of the pore in case of LGC with closed lid (**b**) and open lid (**c**). In case of LGC with closed lid in (**b**), the lid looks flat on the pore covering it whereas in case of LGC with open lid in (**c**), the lid looks slightly tilted owing to its open form. Scale bar: 50 nm. The data is representative of n=2 independent experiment. Source data are provided as a Source Data file.

Supplementary Note 1: Agarose gel electrophoresis analysis of the interaction of LGC-N pore with SUVs under different conditions

Here, two types of purified LGC-N pore samples: LGC-N +Mg (dissolved in 0.5 × TAE buffer containing 10 mM $MgCl_2$) and LGC-N -Mg (dissolved in 50 mM HEPES (pH 7.6) supplemented with 500 mM NaCl), and two cholesterol-modified variants and their incubations with SUVs, were run on 1.5% agarose gel with running buffer 0.5 × TAE buffer containing 10 mM $MgCl_2$ (Supplementary Figure 8a,b) or 1.5% agarose gel with running buffer 0.5 × TAE buffer without 10 mM $MgCl_2$ (Supplementary Figure 8c,d). From the gel images, we can see that all the LGC-N +Mg pores incubated with SUVs showed upshift bands in the gel pockets, suggesting a strong binding of pores with SUVs, no matter with or without cholesterol modifications, so there were non-specific adsorption interactions between the pores without cholesterol modifications and SUVs; while for LGC-N -Mg pores, only the sample run in Mg^{2+} containing gel (Supplementary Figure 8b) showed non-specific binding and the one in Mg^{2+} free gel (Supplementary Figure 8d) showed a clear band shift similar to the pore alone, meaning no binding of the pore with the SUVs. We assume the non-specific binding is caused by Mg^{2+}, either it originating from the LGC-N +Mg pore solution, or from the gel/running buffer, or both, and the divalent cation Mg^{2+} can mediate the adsorption of negatively changed LGC-N pore and the phospholipid membrane. Therefore, it's essential to keep a Mg^{2+} free condition to avoid non-specific interactions between the pores and lipid membrane.

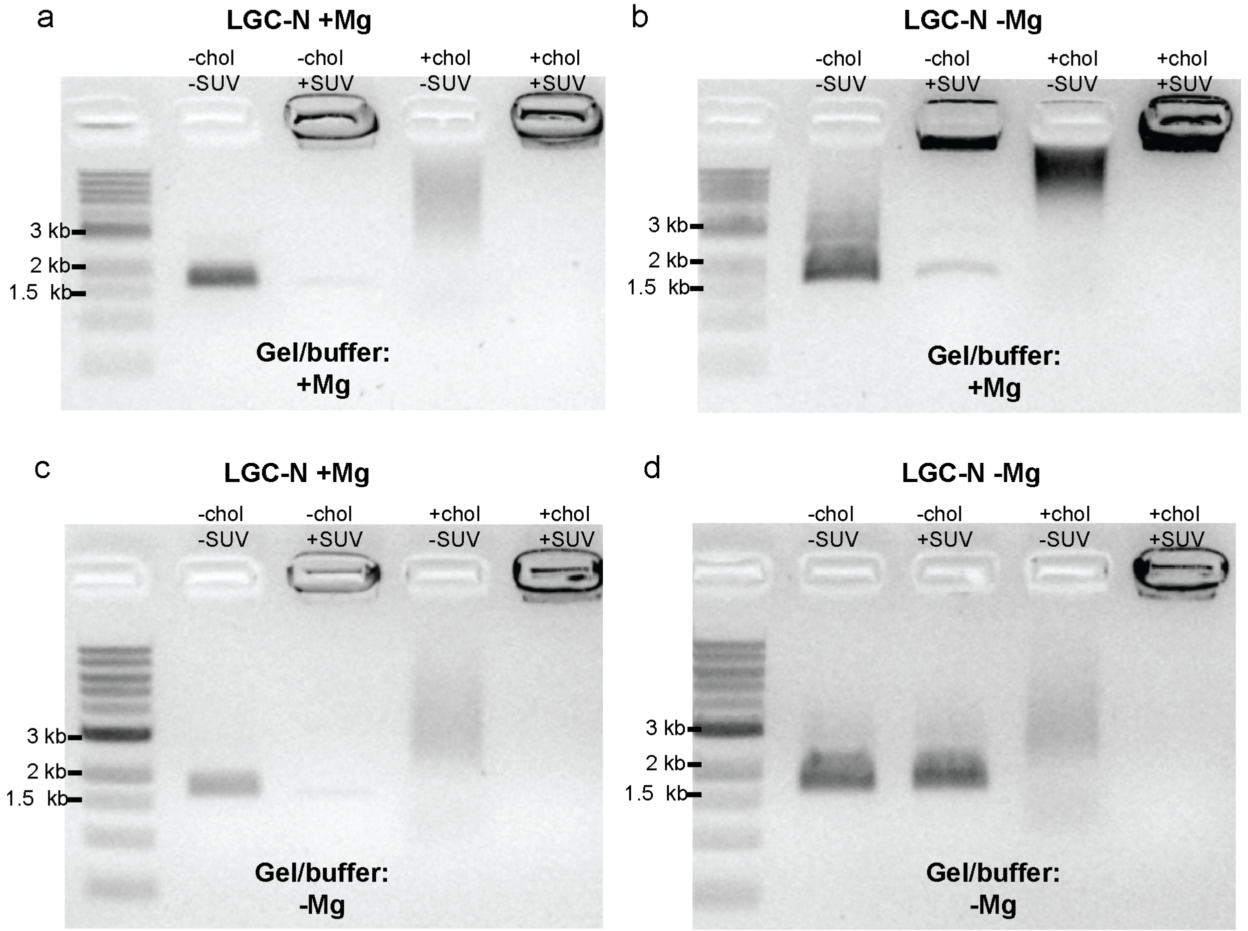

Supplementary Figure 8: Agarose gel analysis of the interaction of LGC-N pore with SUVs under different conditions. The purified LGC-N pores (**a**) LGC-N +Mg dissolved in 0.5 × TAE buffer containing 10 mM $MgCl_2$ and (**b**) LGC-N -Mg dissolved in 50 mM HEPES (pH 7.6) supplemented with 500 mM NaCl were run on 1.5% agarose gel with running buffer 0.5 × TAE buffer containing 10 mM $MgCl_2$; (**c**) LGC-N +Mg and (**d**) LGC-N -Mg were run on 1.5% agarose gel with running buffer 0.5 × TAE buffer without 10 mM $MgCl_2$. The left lanes on each gel were 1 kb DNA ladder, the next four lanes from left to right are the unmodified pore only, unmodified pore incubated with SUVs, cholesterol-modified pore, and cholesterol-modified pore incubated with SUVs. Both the SUVs solution and incubation buffer (50 mM HEPES (pH 7.6) supplemented with 500 mM NaCl) had no added Mg^{2+}. All experiments are repeated at least three times. Source data are provided as a Source Data file.

AFM characterization of LGC in no Mg^{2+} buffer

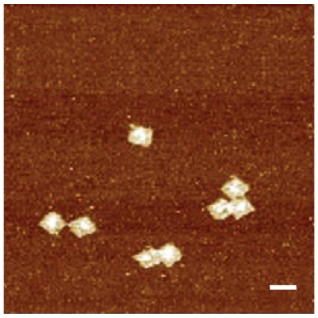

Supplementary Figure 9: AFM image of LGC-C in Mg^{2+} free buffer. The structure is seen to be stable without Mg^{2+} after formation. Scale bar: 100nm. The data is representative of n=2 independent experiments. Source data are provided as a Source Data file.

Interaction of LGC with lipid membrane: large field of view TEM images

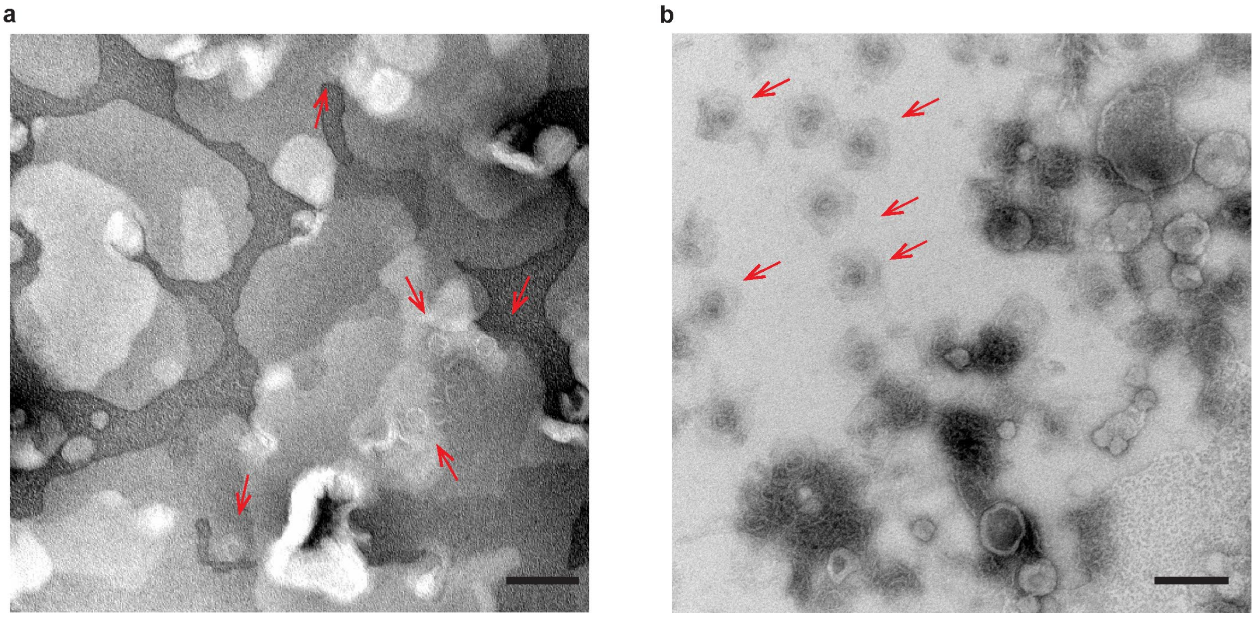

Supplementary Figure 10: Large field of view TEM images showing interaction of no lid LGC (LGC-N) with lipid membrane. LGC-N structures are marked with red arrows. Scale bars: 100 nm. (**a**) POPC-SUVs + LGC-N with cholesterol TEM images show most LGC-N structures on top of the lipid membranes as they strongly interact with the lipid membrane. (**b**) POPC-SUVs + LGC-N without cholesterol TEM images show most LGC structures away from the SUVs due to lack of interaction between unmodified LGC-N structures and lipid membrane. The data is representative of n=2 independent experiments

Confocal images showing proper focus during course of imaging for GUV-atto 633 dye influx assay through Cy3-labelled LGC in main text Fig. 2c-ii

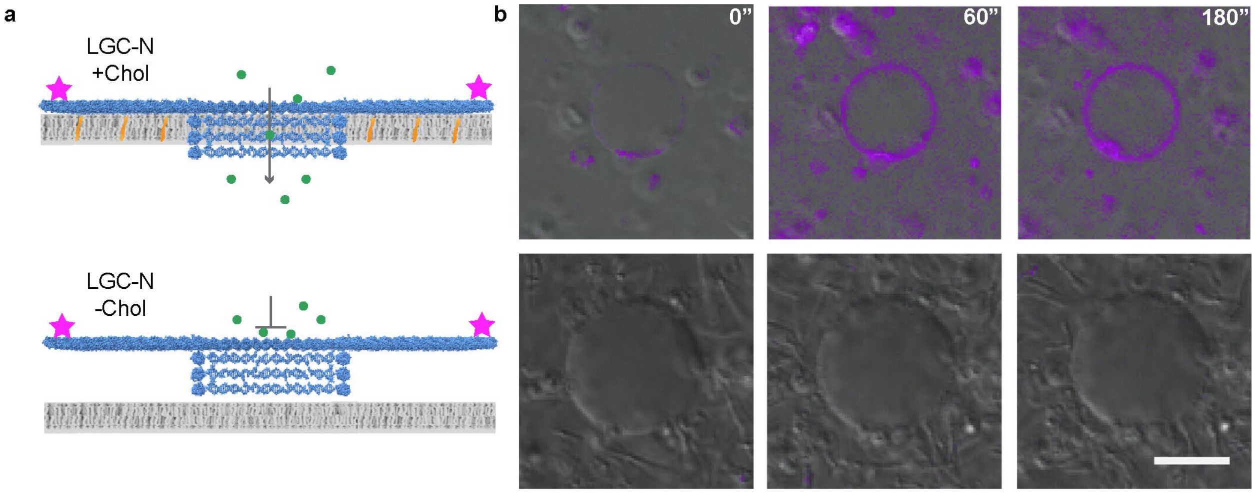

Supplementary Figure 11: (**a**) Scheme and (**b**) Transmitted light (grey) and Cy3 channel (magenta) merged images of GUV influx assay in Fig. 2c-ii in main text, showing that the GUVs remained on focus across the imaging. **Top**: no lid LGC with cholesterol (LGC-N +Chol), **bottom**: no lid LGC without cholesterol (LGC-N -chol). Scale bar: 10 µm. The data is representative of n=3 independent experiments. Source data are provided as a Source Data file.

FRET assay to demonstrate membrane spanning and stable channel formation by cholesterol modified LGC

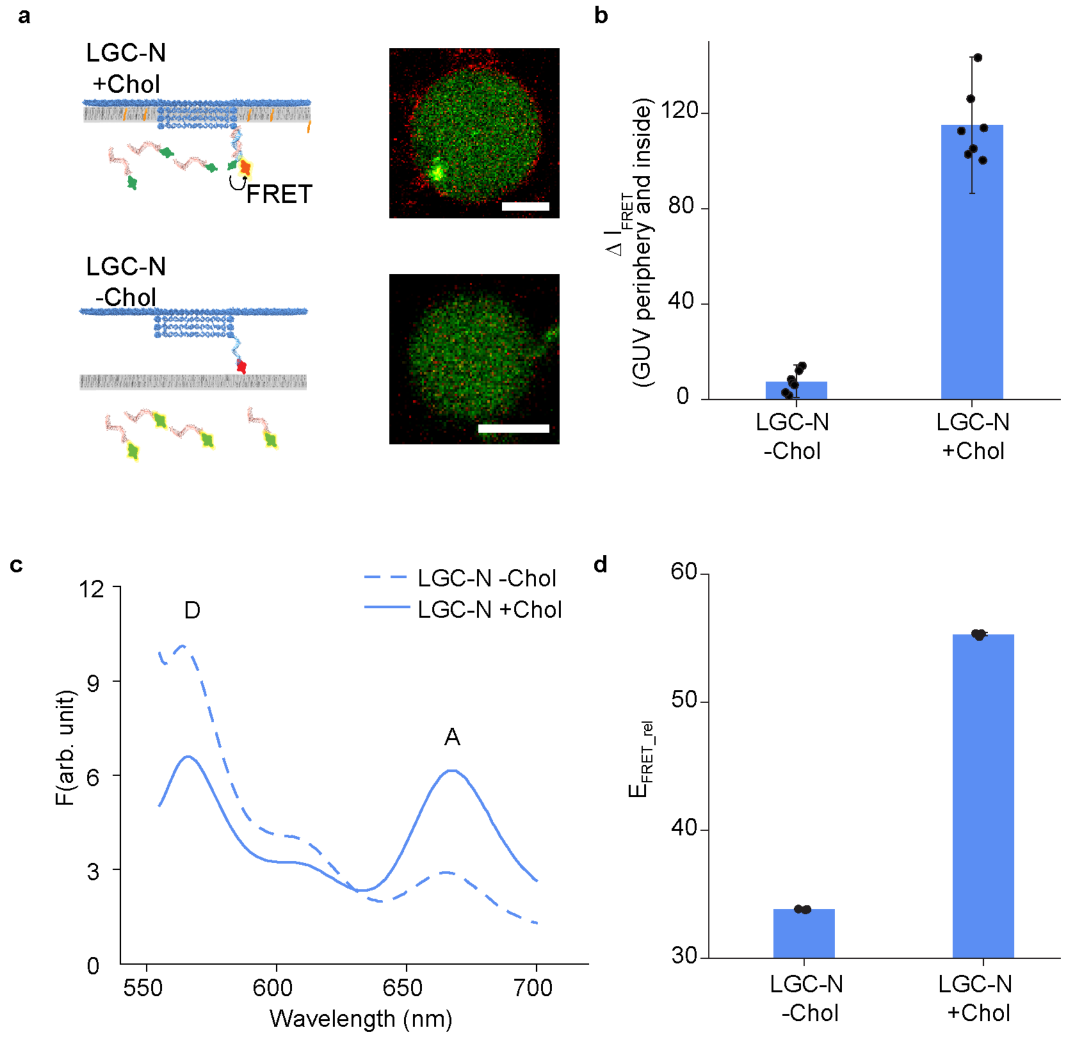

Supplementary Figure 12: FRET measurements for insertion of the nanopore into the lipid bilayer. **a,** Scheme (left panels) and corresponding FRET confocal micrographs (right panels) of no lid pore (LGC-N) bearing a Cy5 labelled probe strand is added to GUVs filled with Cy3 labelled strand complementary to the Cy5 labelled probe. Cholesterol labelled pores span the bilayer, causing hybridization of the Cy5 labelled probe strand of LGC-N with the Cy3 labelled strands inside the GUV (**a, top row**). This results in FRET signal observed as a red perimeter around the GUVs showing that the LGC (+Chol) has inserted into the lipid bilayer and the pore is formed without collapsing. Similar red FRET perimeter is not observed in case of non-cholesterol LGC which cannot insert into the lipid bilayer (**a, bottom row**). FRET confocal micrographs obtained by overlaying the Cy3 donor emission at donor excitation (green) and the Cy5 acceptor emission at donor excitation (red). The data is representative of 3 independent experiments. (Scale bar: 5 µm). **b,** Difference of FRET intensity (Cy5 acceptor emission at Cy3 donor excitation, red) between the perimeter and the inside of the GUV was used to quantify the FRET from 10-13 GUVs across 3 technical replicates observed when the nanopore inserts into the lipid bilayer and error bar shows the standard deviation of mean of the difference in the FRET intensity between the perimeter and inside of the GUV. Cholesterol modified LGC-N shows much higher FRET compared to that of non-cholesterol LGC-N. **c,** Representative ensemble FRET spectra and **d,** corresponding relative FRET efficiency obtained when no lid pore (LGC-N) bearing a Cy5 labelled probe strand is added to GUVs filled with Cy3 labelled strand complementary to the Cy5 labelled probe. (D and A on graph represent fluorescence maxima for donor and acceptor respectively). Cholesterol modified LGC-N shows lower donor fluorescence, higher acceptor fluorescence **(c, solid line)** and high FRET efficiency **(d, LGC-N+Chol).** Non-cholesterol

LGC-N shows higher donor fluorescence, lower acceptor fluorescence **(c, dashed line)** and lower FRET efficiency **(d, Open, LGC-N-chol)**. The FRET efficiency bars and error bars in **d** represents mean relative FRET efficiency and standard deviation of the mean respectively, obtained from three technical replicates. Both the FRET confocal micrographs and the spectral data demonstrate that the LGC is able to successfully insert into the bilayer and maintain the structure without collapsing. Source data are provided as a Source Data file.

Single traces showing kinetics of atto-633 dye/GFP influx into GUVs

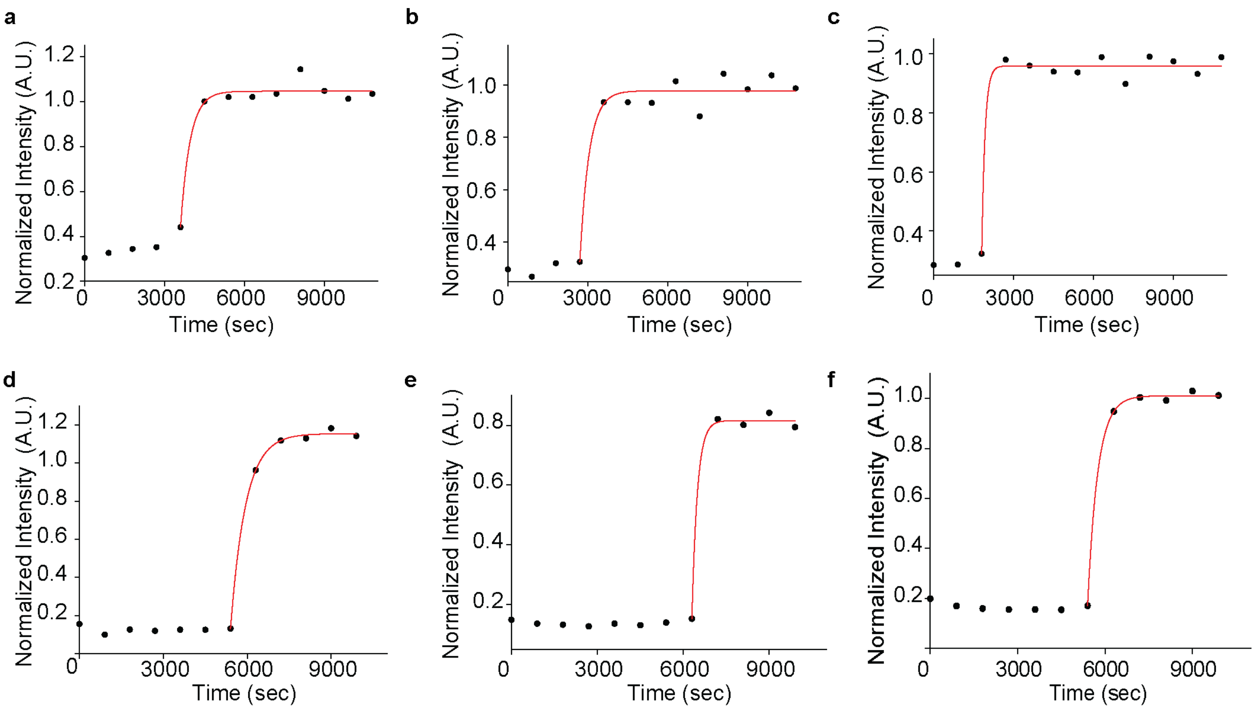

Supplementary Figure 13: Single traces demonstrating the increase of fluorescence inside the vesicles due to the influx of (**a-c**) Atto 633 and **(d-f)** GFP protein through no lid pore LGC-N. The traces are fit using monoexponential model. Source data are provided as a Source Data file.

Supplementary note 2: Kinetics of dye influx

The kinetics of dye influx inside the GUVs was estimated using the Fick's law following calculation by Krishnan et al[3] (supplementary info P. 29). As per the calculation shown there, the rate of Atto633 dye diffusion into the GUV interior through LGC is given by

$$n_i(t) = n_o(1 - e^{-k_o t}) \quad (1)$$

Where, $n_i(t) =$ dye concentration inside the GUVs at time t;

$n_o(t) =$ dye concentration outside the GUVs

$k_o =$ first order dye diffusion rate constant $= \frac{DA}{LV}$;

Where, D = diffusion coefficient of the dye, A = Cross-sectional area of the nanopore, L = Length of the nanopore and V = internal volume of the GUVs.

The value of diffusion coefficient of atto dyes[4,5] has been found close to D = 426 $\mu m^2/sec$. Now putting the values A = 416 nm^2, L = 10nm from the nanopore design and V = 117.1 μm^3 , derived from the average diameter of GUVs in the confocal measurements as 6.07 μm. This yields $k_o = 0.015\ s^{-1}$. Now the time required for dye influx through a single nanopore

$$\tau_{single} = \frac{1}{k_o} = \frac{1}{0.015\ Sec^{-1}} = 6.6\ Sec. \quad (2)$$

In our actual observation (Supplementary Fig. 13) we see much slower diffusion rates. This is contrasting to the previous reports, where the observed for dye diffusion was always faster compared to the theoretically predicted value. The observed rate of dye diffusion through DNA

nanopore is a combination of several inter-dependent phenomena, i.e.- i) nanopore binding to the membrane, ii) reorientation of its stem in the bilayer to form a channel and finally iii) the diffusion of the dye through the pore. The nanopore binding to membrane is strongly governed by the number of cholesterol molecules, ionic concentration, temperature etc. While the reorientation step is supposedly the slowest and rate determining step, that depends on the flexibility of the cholesterol bearing segments of the nanopore. The final step of dye diffusion is a function of the size of dye molecule, medium viscosity, temperature, concentration gradient and pore dimensions. We attribute the faster flux in case of our LGC pore compared to the previous pores due to the placement of large number of cholesterol anchors to help the step (i). The cholesterol-bearing one layer origami plate being flexible enough for rapid re-orientation of the pores, probably led to faster insertion kinetics compared to the previous origami pores that always placed the cholesterol molecules in a rigid framework, thereby slowing the reorientation step. Interestingly, the dye diffusion rate in LGC pore was ~5 times slower compared to the theoretically calculated time required for diffusion (Supplementary Fig. 13). This might have resulted as contribution of the wider pore dimension of LGC toward the diffusion rate was overcome by the reorientation step that governs the overall observed rate of dye influx. Further detailed investigation is required to decouple the absolute contributions of the three steps in the insertion of hydrophobically modified DNA nanostructures into lipid bilayer.

No cap DNA plate as control to show that the cholesterol modifications are not disturbing the membrane or making it leaky

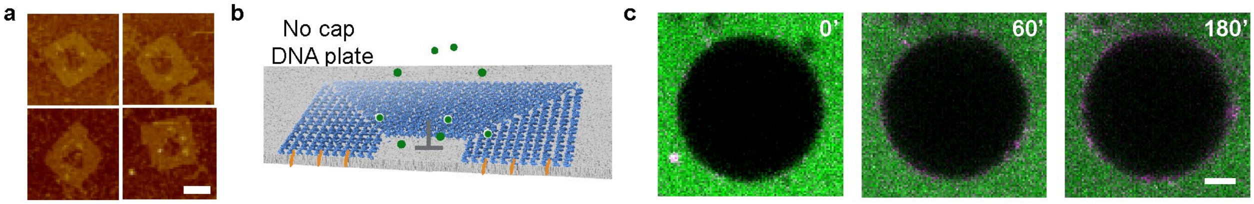

Supplementary Figure 14: (**a**) AFM images of the no cap DNA plate. Compared to the AFM images of the original LGC, the region around the hole in the middle of this structure has the same height as the rest of the structure showing the absence of the pore forming stem region in this structure. (Scale bar: 50 nm). The data is representative of n=3 technical repeats. (**b**) Schematic of the no cap DNA plate structure inserted into the lipid bilayer of a GUV. Due to the absence of the pore, dye influx should not happen. (**c**) The cy3 labelled no cap DNA plate is able to insert into the GUV as seen by the magenta around the GUVs. But due to the lack of pore formation, no dye influx is observed even after 3 hrs. This shows that the cholesterol insertion itself is not causing the dye influx but the ability of the structure itself to form a pore in the membrane is leading to transport of dye across the membrane. (Scale bar: 5µm). The data is representative of n=2 independent experiments. Source data are provided as a Source Data file.

Negative control no-cap DNA plate

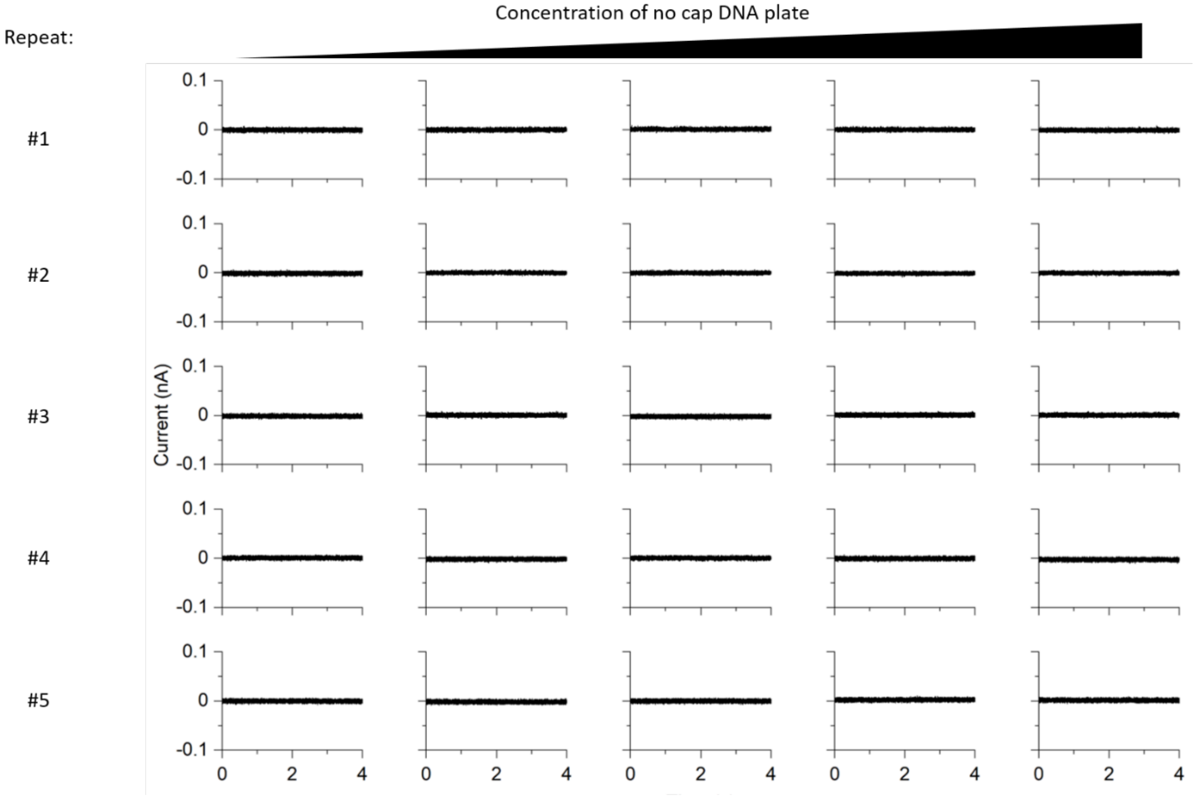

Supplementary Figure 15: A no cap DNA plate does not cause pore forming currents. Single-channel current recordings of DPhPC membranes after the addition of increasing concentrations of LGC-N without the pore forming cap region. Even after high concentrations of the DNA plate have been added, no pore forming currents were recorded.

Conductance states of LGC-N

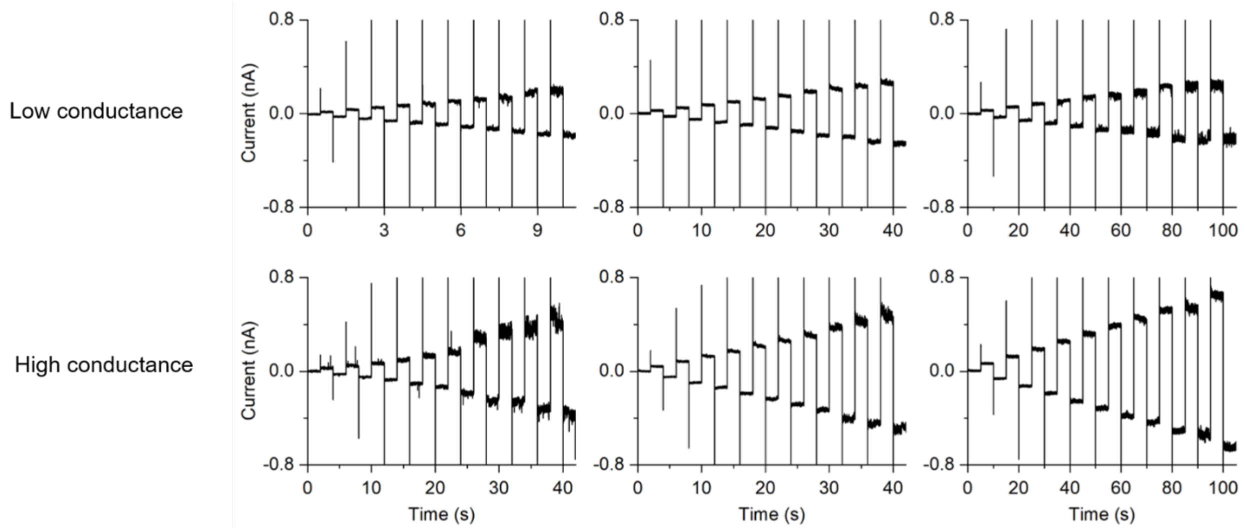

Supplementary Figure 16: LGC-N has high and low conductance states. Example electrophysiological traces showing six individual pore insertions of LGC-N, three low conductance pores (<5 nS) and three high conductance pores (>5 nS). Example traces measured by alternating voltage in +/- 10 mV steps starting at 0 mV and ending at -100 mV.

Cyclic opening and closing of the lidded LGC Negative control with lid opening using mismatch opening key

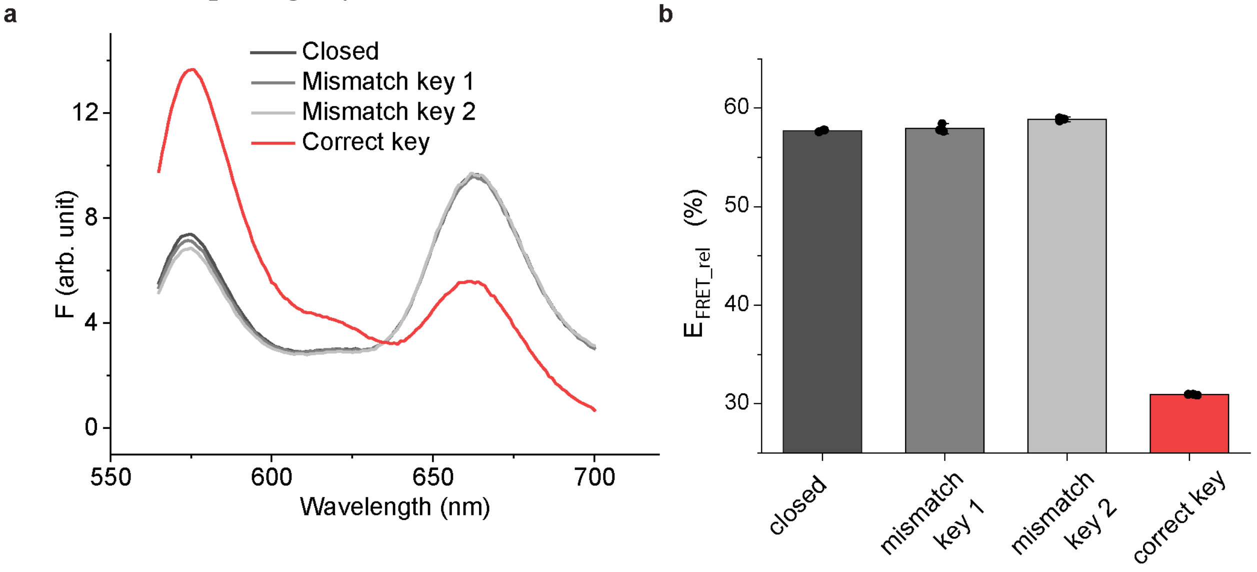

Supplementary Figure 17: (**a**) FRET spectra and (**b**) corresponding FRET efficiencies - for opening of LGC with closed lid by mismatch key and correct key. FRET spectra and FRET efficiencies of the LGC with closed lid with two different sets of mismatch keys behave similar to closed lid structure only, i.e. – low donor fluorescence at $\lambda_{max}^{Cy3} = 564nm$ and higher acceptor fluorescence at $\lambda_{max}^{Cy5} = 670nm$ (a-i; black, light grey and dark grey curves) and higher FRET efficiency, a-ii; black, dark grey and light grey bars). Whereas the same with correct opening key (Fig.1 - red) leads to opening of the lid and hence shows higher donor fluorescence at $\lambda_{max}^{Cy3} = 564nm$ and lower acceptor fluorescence at $\lambda_{max}^{Cy5} = 670nm$ (**a, solid red curve**) and higher FRET efficiency (**b, red bar**). The FRET efficiency bars and error bars represents mean relative FRET efficiency and standard deviation of the mean respectively, obtained from three technical replicates. Source data are provided as a Source Data file.

Supplementary note 3: Kinetics of lid opening by FRET

The key mediated opening of the LGC is an intermolecular reaction, and so it was analyzed as a second order reaction[6]. For the reaction shown below, the rate constant of the forward reaction is k. Once again, the backward reaction can be neglected because the free energy of hybridization precludes loss of the displacement strand.

$$Lid_{closed} + Key \rightarrow Lid_{open} \tag{3}$$

At any given time point t, the rate of the reaction can be expressed as –

$$r = -\frac{d[C]}{dt} = \frac{d[P]}{dt} = k[C]_t[F]_t \tag{4}$$

Where at any given time point t,

$[C]_t = conc\ of\ Lid_{closed}$

and, $[F]_t = conc\ of\ Key$

Now, if

$[C]_0 = conc\ of\ Lid_{closed}\ at\ t = 0\ and\ [P] = conc\ of\ the\ product, i.e. Lid_{open}\ at\ time\ t$

Then, at any given point the total amount of closed form and the open form of the Lid is the same as the starting concentration of closed form, i.e. $[C]_0$.

Therefore,

$$[C]_t + [P] = [C]_0$$

i.e.

$$[C]_t = [C]_0 - [P] \tag{5}$$

and similarly, for the target -

$$[F]_t + [P] = [F]_0$$

i.e.

$$[F]_t = [F]_0 - [P] \tag{6}$$

Now, replacing the value of $[C]_t$ and $[F]_t$ from equation (4):

$$\frac{d[P]}{dt} = k([C]_0 - [P])([F]_0 - [P]) \tag{7}$$

If we start with n fold of the target compared to the Lid_{closed}, then

$$[F]_0 = n[C]_0 \tag{8}$$

Hence, replacing the value of $[F]_0 = n[C]_0$ in equation (7) and integrating from t = 0 to t gives -

$$\int_0^t kdt = \int_0^{[P]} \frac{d[P]}{([C]_0 - [P])(n[C]_0 - [P])} \tag{9}$$

Now, by simplifying $[C]_0 = C \; and \; [P] = x$, we get -

$$\int_0^{[P]} \frac{d[P]}{([C]_0 - [P])(n[C]_0 - [P])} = \int_0^x \frac{dx}{(C - x)(n.C - x)} \tag{10}$$

$$\frac{1}{(C - x)(n.C - x)} = \frac{A}{(C - x)} + \frac{B}{(n.C - x)} = \frac{(A.n + B)C - (A + B)x}{(C - x)(n.C - x)}$$

Therefore, $(A.n + B)C - (A + B)x = 1$

Therefore,

$$A + B = 0; \; (A.n + B)C = 1$$

$$A = -B = \frac{1}{(n-1)C}$$

Hence, from equation (9) and (10)

$$\int_0^{[P]} \frac{d[P]}{([C]_0 - [P])(n[C]_0 - [P])} = \frac{1}{(n-1)C}\left(\int_0^x \frac{dx}{(C-x)} - \int_0^x \frac{dx}{(n.C-x)}\right)$$

$$= \frac{1}{(n-1)C}([-\ln(C-x)]_0^x - [-\ln(n.C-x)]_0^x)$$

$$= \frac{1}{(n-1)C}(lnC - \ln(C-x) + \ln(n.C-x) - \ln(n.C)) \qquad (11)$$

Putting back the values $[C]_0 = C \; and \; [P] = x$ in equations (9) and (11), we get –

$$\frac{1}{(n-1)[C]_0}(ln[C]_0 - \ln([C]_0 - [P]) + \ln(n.[C]_0 - [P]) - \ln(n.[C]_0)) = kt$$

$$(ln[C]_0 - \ln([C]_0 - [P]) + \ln(n[C]_0 - [P]) - \ln(n[C]_0)) = kt(n-1)[C]_0$$

Replacing $[C]_0 - [P] = [C]_t$ from equation (5)

$$(ln[C]_0 - \ln[C]_t + \ln(n[C]_0 - [P]) - \ln(n[C]_0) = kt(n-1)[C]_0$$

$$ln\frac{n[C]_0 - [P]}{[C]_t} - ln\frac{n[C]_0}{[C]_0} = kt(n-1)[C]_0 \qquad (12)$$

Putting $[P] = [C]_0 - [C]_t$ into equation (12)

$$ln\frac{(n-1)[C]_0 + [C]_t}{[C]_t} = \ln(n) + kt(n-1)[C]_0$$

$$ln\left[\frac{(n-1)[C]_0}{[C]_t} + 1\right] = \ln(n) + kt(n-1)[C]_0$$

$$\frac{(n-1)[C]_0}{[C]_t} = \exp[\ln(n) + kt(n-1)[C]_0] - 1$$

$$= \exp[\ln(n)].\exp[kt(n-1)[C]_0] - 1$$

$$\frac{(n-1)[C]_0}{[C]_t} = \exp[\ln(n)].\exp[kt(n-1)[C]_0] - 1 = \mathrm{n}.\exp[kt(n-1)[C]_0] - 1$$

$$\frac{[C]_t}{[C]_0} = \frac{n-1}{n.\exp[kt(n-1)[C]_0] - 1} \qquad (13)$$

The next step is to relate the equation above to the experimental data we collected. For each kinetic curve, at time 0, after time t, and at the end of the reaction (t goes to ∞), the normalized fluorescence intensities are I_0, I_t, and I_∞, respectively. Hence by proportionality –

$$\frac{[C]_t}{[C]_0} = \frac{I_\infty - I_t}{I_\infty - I_0} \qquad (14)$$

Thus, equating equations (13) and (14), we obtain –

$$\frac{I_\infty - I_t}{I_\infty - I_0} = \frac{n-1}{n.\exp[kt(n-1)[C]_0] - 1}$$

$$I_t = I_\infty - (I_\infty - I_0).\frac{n-1}{n.\exp[kt(n-1)[C]_0] - 1} \qquad (15)$$

The equation (15) is used to fit the normalized kinetic curve with time to obtain the rate constant k.

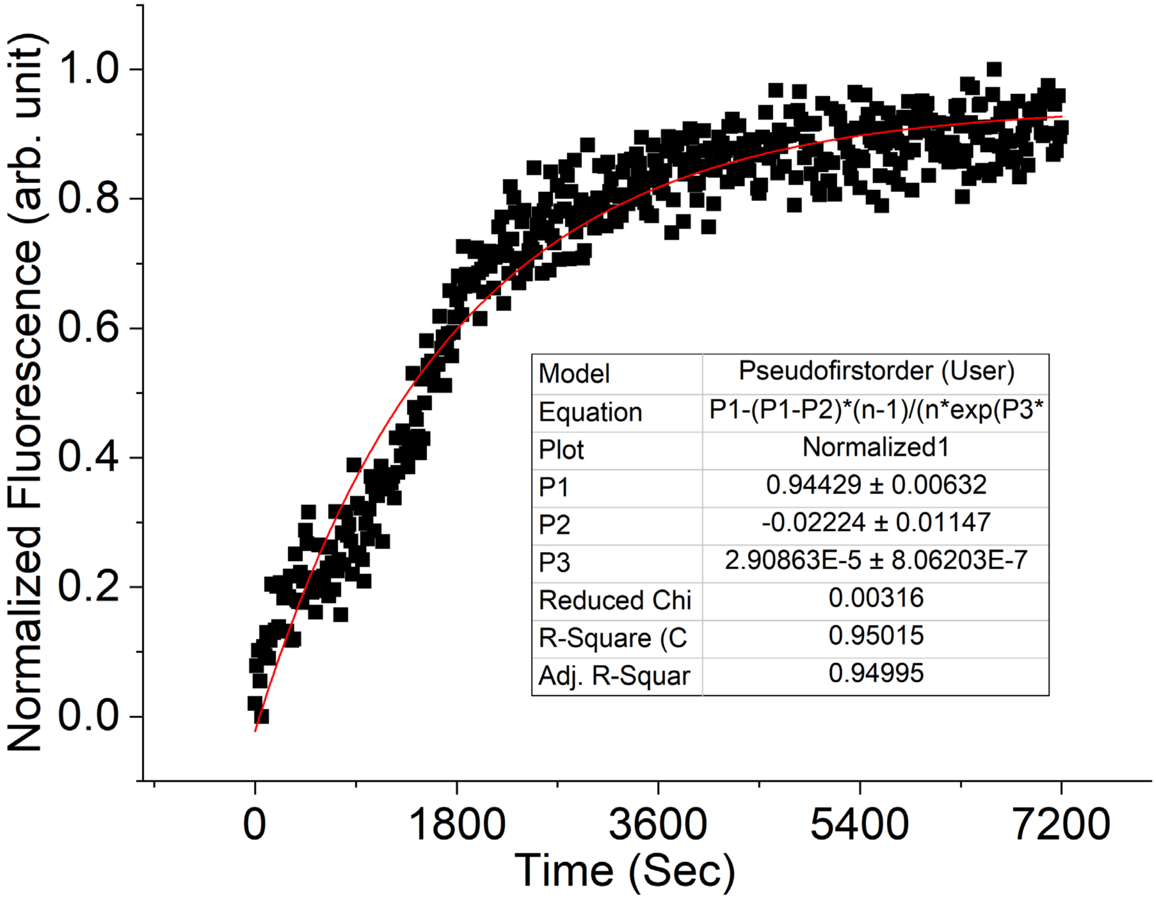

Supplementary Figure 18: Kinetics of lid opening. Data is fitted with second order kinetic rate equation (R^2 = 0.94995) to obtain Rate constant = 1940 ± 50 M^{-1} S^{-1}. Source data are provided as a Source Data file.

Negative controls for atto-633 dye influx experiments in main text Fig. 3b-ii: LGC with closed and opened lid without cholesterol modification

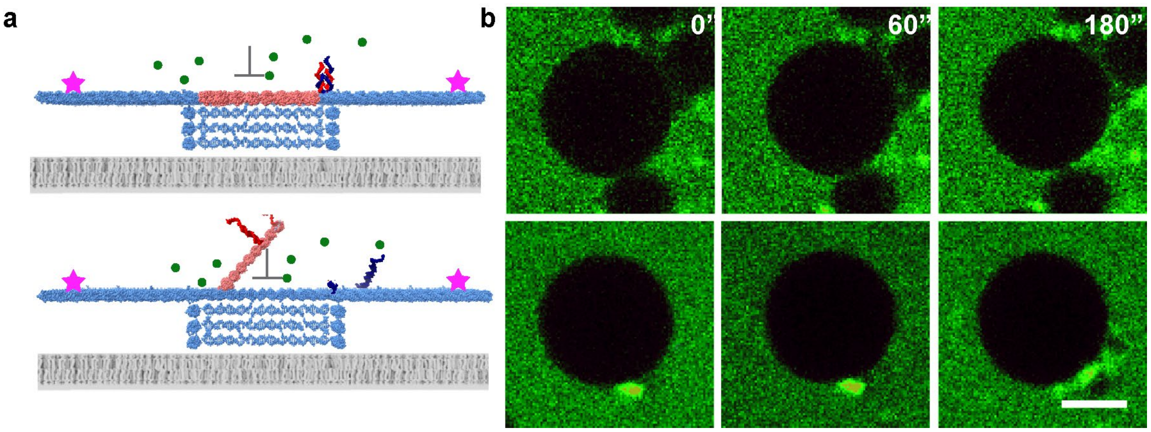

Supplementary Figure 19: Negative control experiments for Fig. 3b-ii in the main text. (**a**) Cy3 labelled LGC structures (magenta) without cholesterol modification don't bind to the GUV membrane. (**b**) Unmodified LGC with closed lid (top panels) or with opened lid (bottom panels) does not show any influx of atto-633 dye (green) into GUVs. Scale bar- 10 µm. The data is representative of n=3 independent experiments. Source data are provided as a Source Data file.

Negative control influx assays using mismatch key

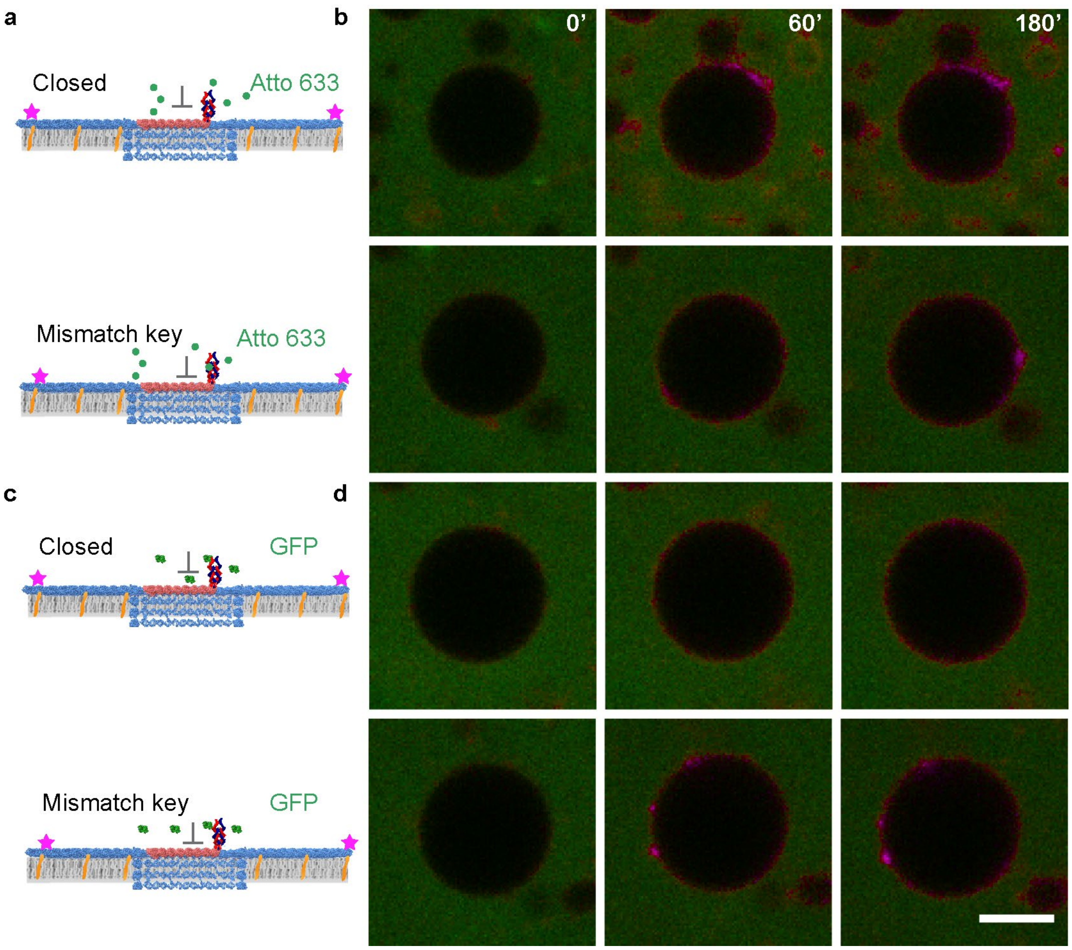

Supplementary Figure 20: (**a**) Scheme and (**b**) confocal images of Atto 633 influx in GUVs showing – top: Cy3 (magenta) and cholesterol labelled LGC with closed lid inserts in GUV but prevents Atto 633 influx. Bottom – addition of mismatch key does not open the LGC lid and thus no Atto-633 influx is observed unlike that in the case of correct opening key in Fig. 3e in the main text. The data is representative of n=3 independent experiments.(**c**) Scheme and (**d**) confocal images of GFP influx in GUVs showing – top: Cy5 (magenta) and cholesterol labelled LGC with closed lid inserts in GUV but prevents GFP influx. Bottom – addition of mismatch key does not open the LGC lid and thus no GFP influx is observed unlike that in the case of correct opening key in Fig. 4b in the main text. The data is representative of n=3 independent experiments. Scale bar: 10 µm. Source data are provided as a Source Data file.

Two dye influx to show dynamic closing of the lid

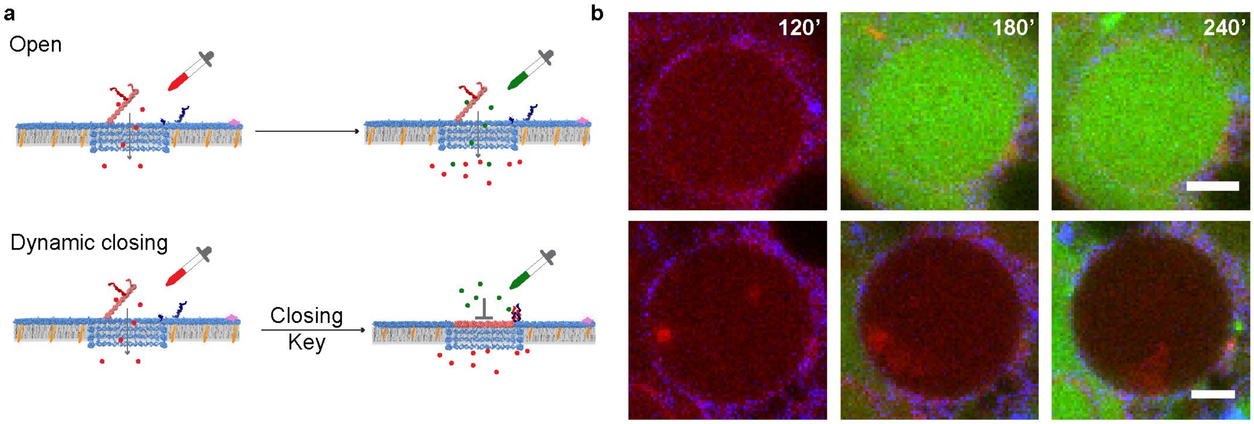

Supplementary Figure 21: (a) Scheme and **(b)** Fluorescence microscopy images to show dynamic closing using two dyes. First a red dye (Atto 633) is added to the LGC-O. Due to the open pore the dye is able to influx into the vesicle. Later the closing key is added only to the bottom panel. In the top panel the pore remains open while in the bottom panel the closing key renders the pore closed. A green dye (Atto 488) was then added to both samples. The dye only influxes into the vesicle where the pore is open. The data is representative of n=2 independent experiments. (Scale bar: 5µm).

All-point histogram analysis for single channel current recordings of open lid pore

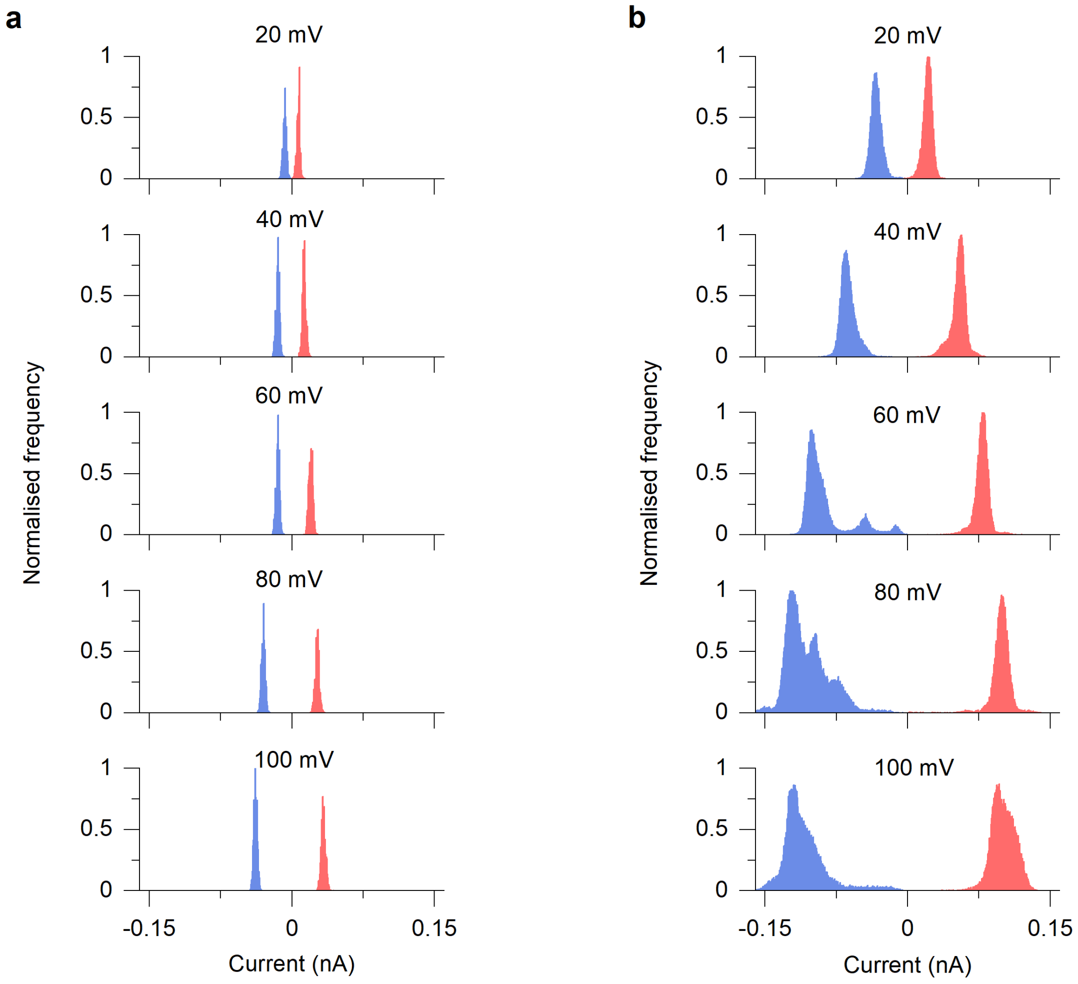

Supplementary Figure 22: All point histogram analysis of (**a**) LGC-closed and (**b**) LGC-open at positive (red) and negative (blue) 20, 40, 60, 80 and 100 mV. Source data are provided as a Source Data file.

Noise comparison of LGC-C and LGC-O

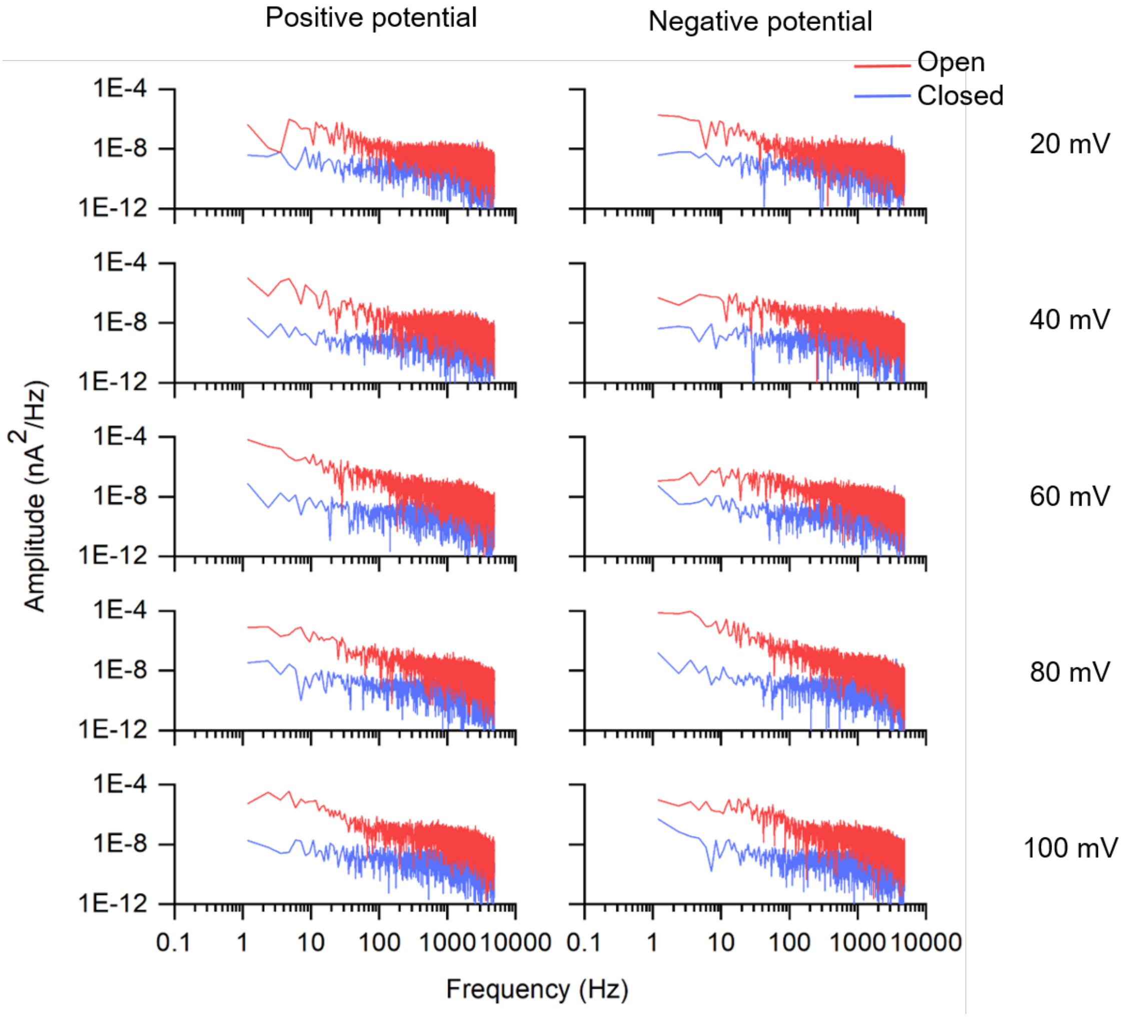

Supplementary Figure 23: LGC-O has a greater noise profile in comparison to LGC-C. Power spectrum analysis of LGC-C (blue, closed) and LGC-O (red, open) at positive and negative potentials ranging from 20 mV to 100 mV at 20 mV intervals. Source data are provided as a Source Data file.

Noise comparison of LGC-O at positive and negative potentials

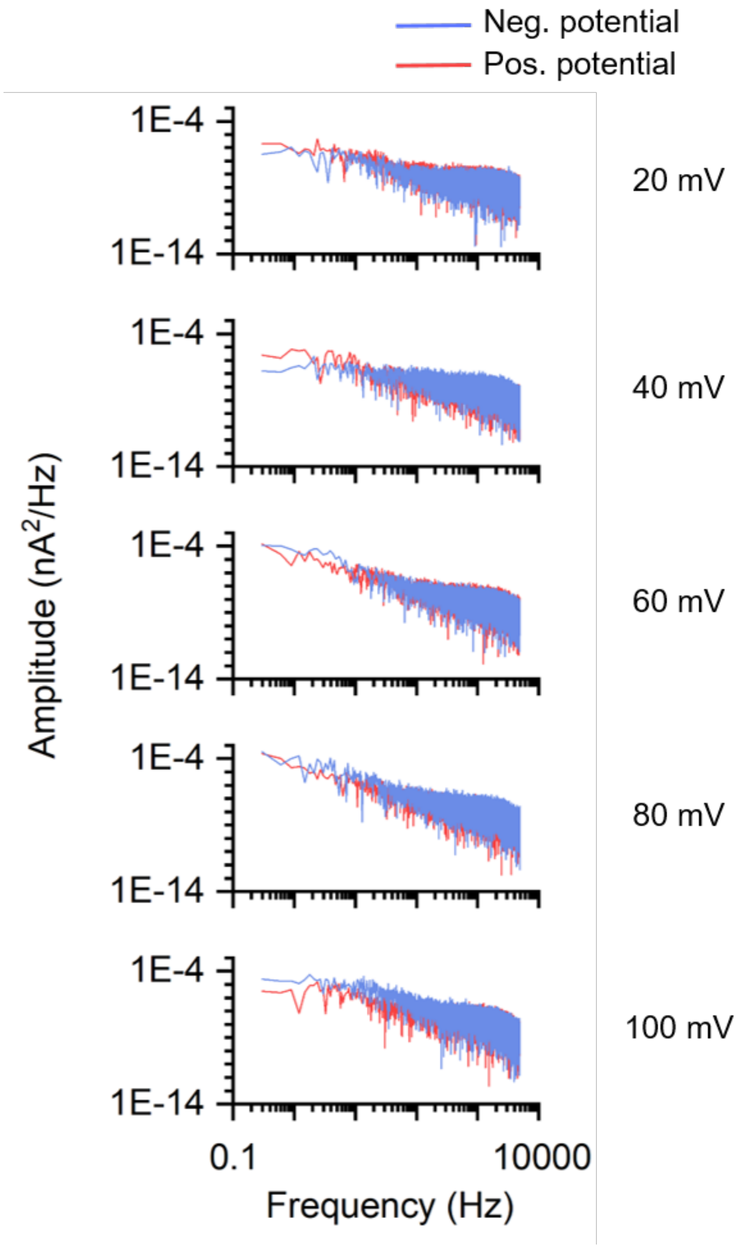

Supplementary Figure 24: LGC-O has more noise at negative potential. Power spectrum analysis of LGC-O at negative (blue) and positive (red) potentials, ranging from 20 mV to 100 mV at 20 mV intervals. Source data are provided as a Source Data file.

Electrophysiological characterization of lid opening-closing

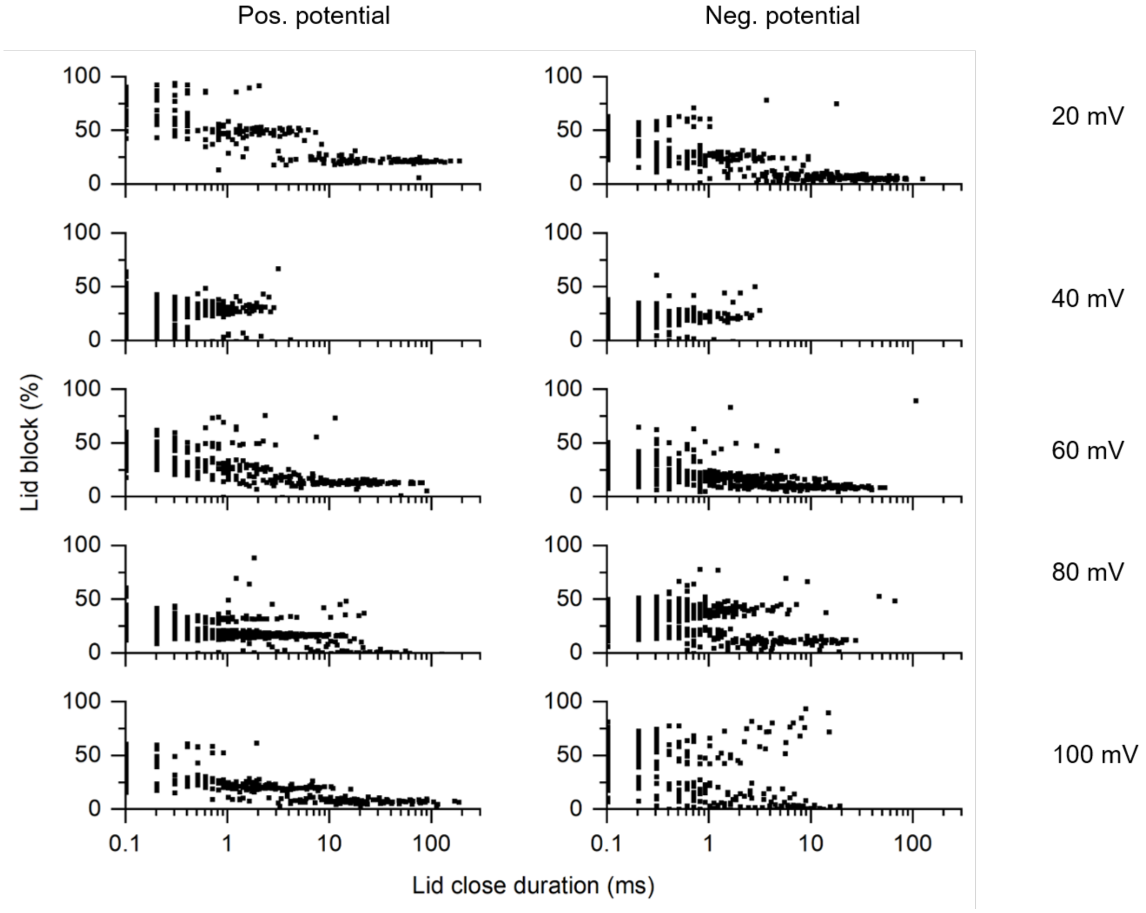

Supplementary Figure 25: LGC-O has a higher frequency of gating effects at negative potentials. Scatter plots of lid closure time compared to the percentage block in current of closure events at positive and negative membrane potentials ranging from 20 mV to 100 mV in 20 mV steps. Source data are provided as a Source Data file.

Electrophysiological characterization of lid opening-closing

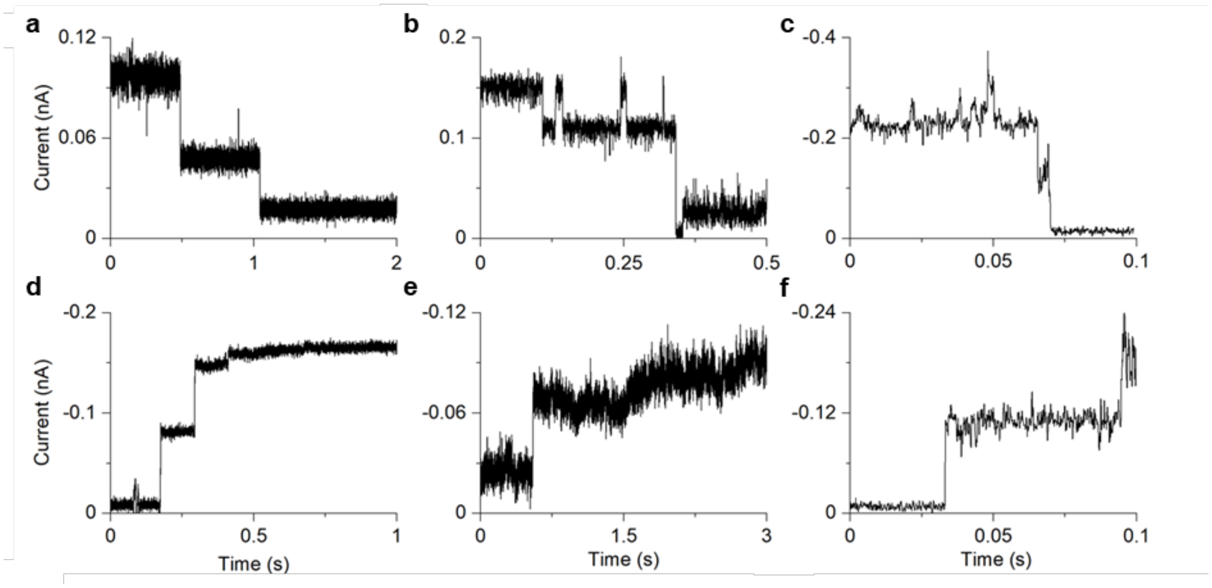

Supplementary Figure 26: **(a-c)** Dynamic closing of open DNA pores at 10 mV (**a**), 50 mV (**b**) and -50 mV (**c**). Closing DNA strands added at a concentration of 15 nM to the buffer solution at 37°C. (**d-f)** Dynamic opening of closed DNA pores at -10 mV (**d**), -20 mV (**e**) and -50 mV (**f**). Opening DNA strands added at a concentration of 15 nM to the buffer solution at 37°C.

GFP influx through LGC with no lid (LGC-N)

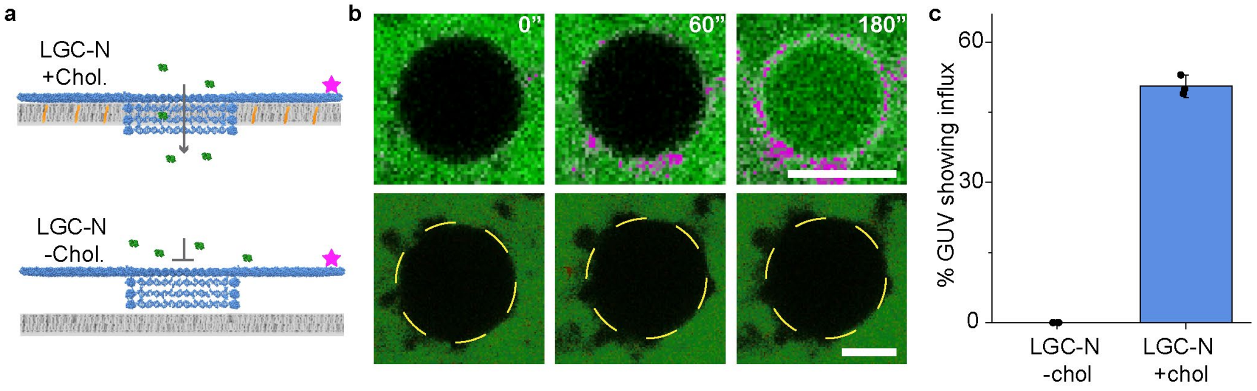

Supplementary Figure 27: (**a**) GUV dye-influx assay scheme and (**b**) their respective time series confocal images at the given intervals with Cy5-labelled LGC-N (magenta) and GFP (green). **Top**: Cholesterol modified LGC-N (LGC-N +Chol) readily interacts with bilayer (magenta circle around GUVs) and their insertion leads to influx of the atto-633 dye inside the GUV interior. **Bottom**: LGC without cholesterol modification (LGC-N -chol) does not interact with bilayer (no magenta circle around GUV) or insert into the GUV, showing no dye influx over the course of 3 hours. The data is representative of n=3 independent experiments. Scale bar: 10 µm. (**c**) Bar plot showing percentage of GUVs showing a filled interior after 3 hours. Data shows average percentage of influx and error bars show standard deviation of mean percentage influx counted from 3 independent experiments across n = 52 GUVs in case of LGC-N + Chol and n = 124 for LGC-N-Chol. Source data are provided as a Source Data file.

Negative controls for GFP influx experiments in main text Fig. 4a-ii: LGC with no lid, closed lid and opened lid without cholesterol modification

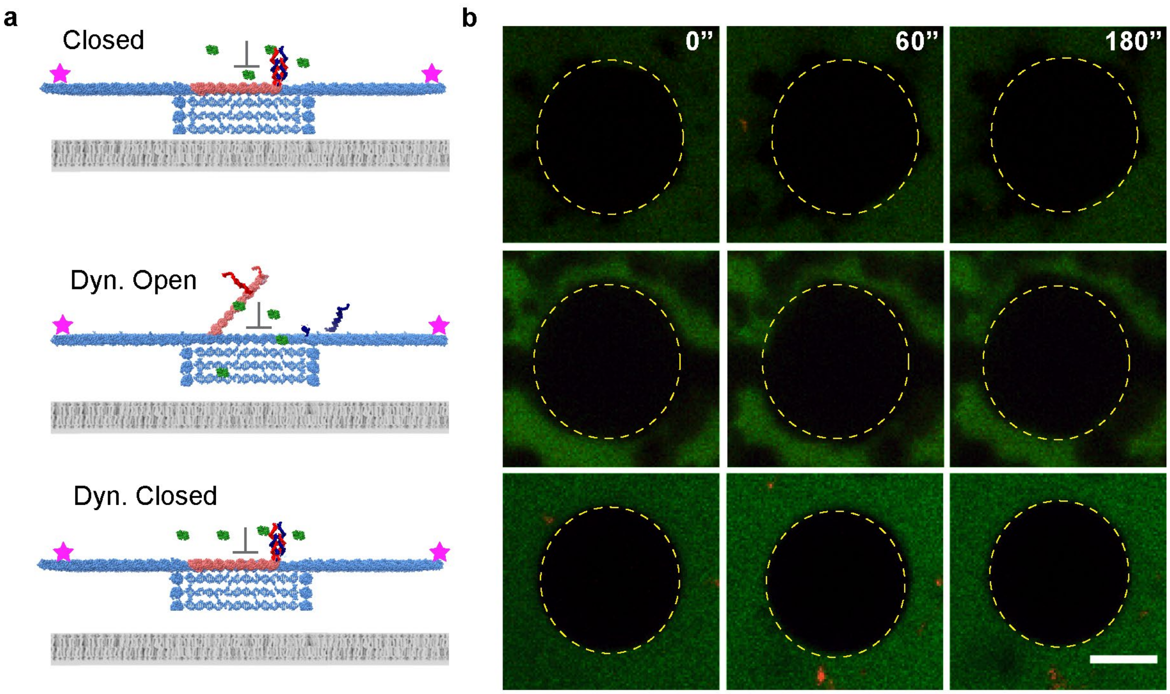

Supplementary Figure 28: Negative control experiments for main text Fig. 4a-ii. (**a**) Cy5 labelled LGC structures without cholesterol modification don’t bind to the GUV membrane. (**b**) GUV influx assay with non-cholesterol LGC, no lid (**top row**), with closed lid (**middle row**) or with opened lid (**bottom row**) does not show any influx of GFP into GUVs. The data is representative of n=3 independent experiments. Scale bar: 10 µm. Source data are provided as a Source Data file.

Negative control to show size dependent transport

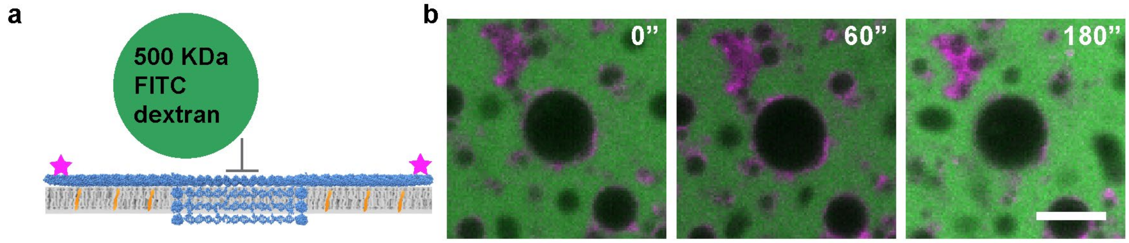

Supplementary Figure 29: Size-selective transport through nanopore. Cy3 (magenta) and cholesterol labelled nanopore without lid inserts through the bilayer but a large molecule such as 500 kDa FITC-Dextran (green) cannot pass through the nanopore. The data is representative of n=3 independent experiment. Scale bar: 10 µm. Source data are provided as a Source Data file.

Cargo release through LGC

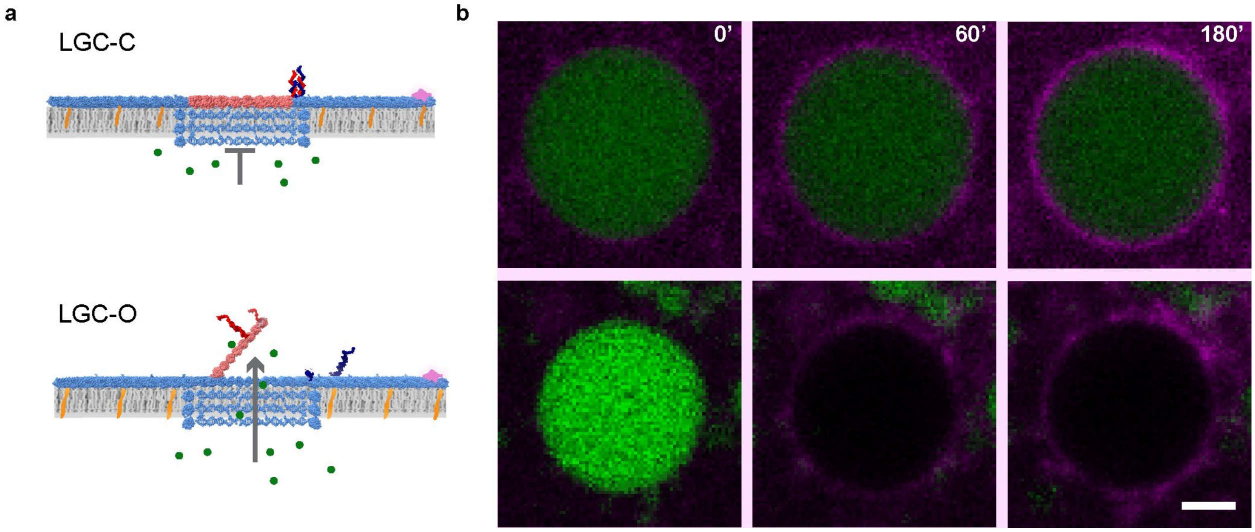

Supplementary Figure 30: (**a**) Schematic of LGC inserted into the lipid membrane of a GUV loaded with cy3 labelled DNA strand (8 nucleotide long). On insertion of LGC-C, the cargo inside the GUV cannot be released outside as the channel is blocked (**top**) but in the open state the cargo can be released to the outside (**bottom**). (**b**) The cy3 labelled LGC-N (magenta) has inserted around the GUV and the cargo inside of the GUV (green), stays inside when the LGC is in closed state. But after the LGC is opened using the key, the cargo is able to leave through the pore. This shows that the structure can potentially be used in drug delivery. The data is representative of n=2 independent experiments. Scale: 5µm. Source data are provided as a Source Data file.

Rate of influx%

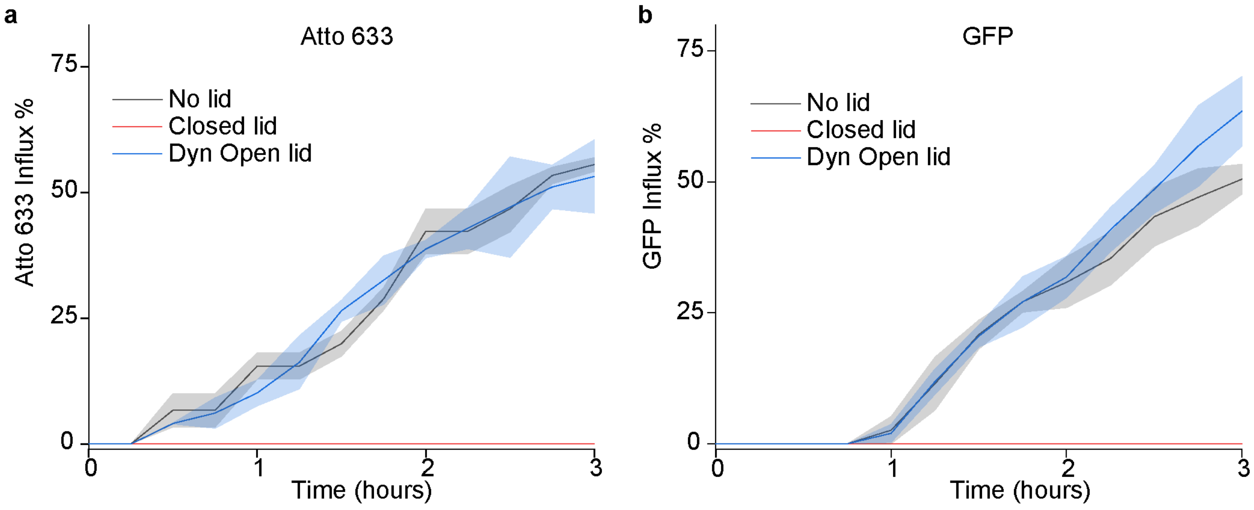

Supplementary Figure 31: Rate of influx % for (**a**) Atto-633 dye and (**b**) GFP protein. Although atto-633 and GFP differs in hydrodynamic diameter, both show similar rate of influx %, perhaps because the LGC pore being large enough compared to both Atto 633 as well as GFP. Moreover, the no lid and dynamically opened lid LGC pores show similar rate of influx % whereas the closed lid pore showing no influx % indicates that the open lid is equivalent to no lid in terms of passage of molecules through the LGC channel. Lines represent average fraction of GUVs (%) that had full influx of Atto-633 dye or GFP protein and grey or blue shades represent standard deviation of the mean obtained from n = 156 GUVs. Source data are provided as a Source Data file.

Analysis of the interaction of trypsin with LGC-C

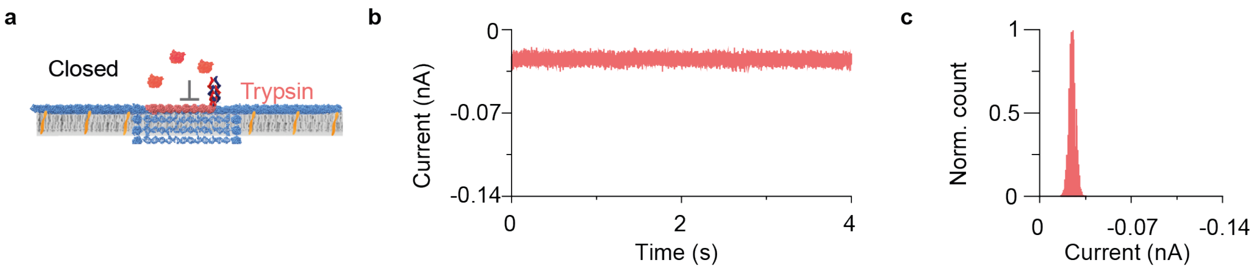

Supplementary Figure 32: (**a**) Trypsin does not translocate through LGC-C. Schematic illustration of LGC-C, with the lid impeding the translocation of trypsin. (**b**) Example trace after addition of 6.6 µM trypsin shows no translocation events. (**c**) Current histogram shows a single peak, indicative of a single current level. Source data are provided as a Source Data file.

Increasing trypsin concentration leads to increased translocation events per second

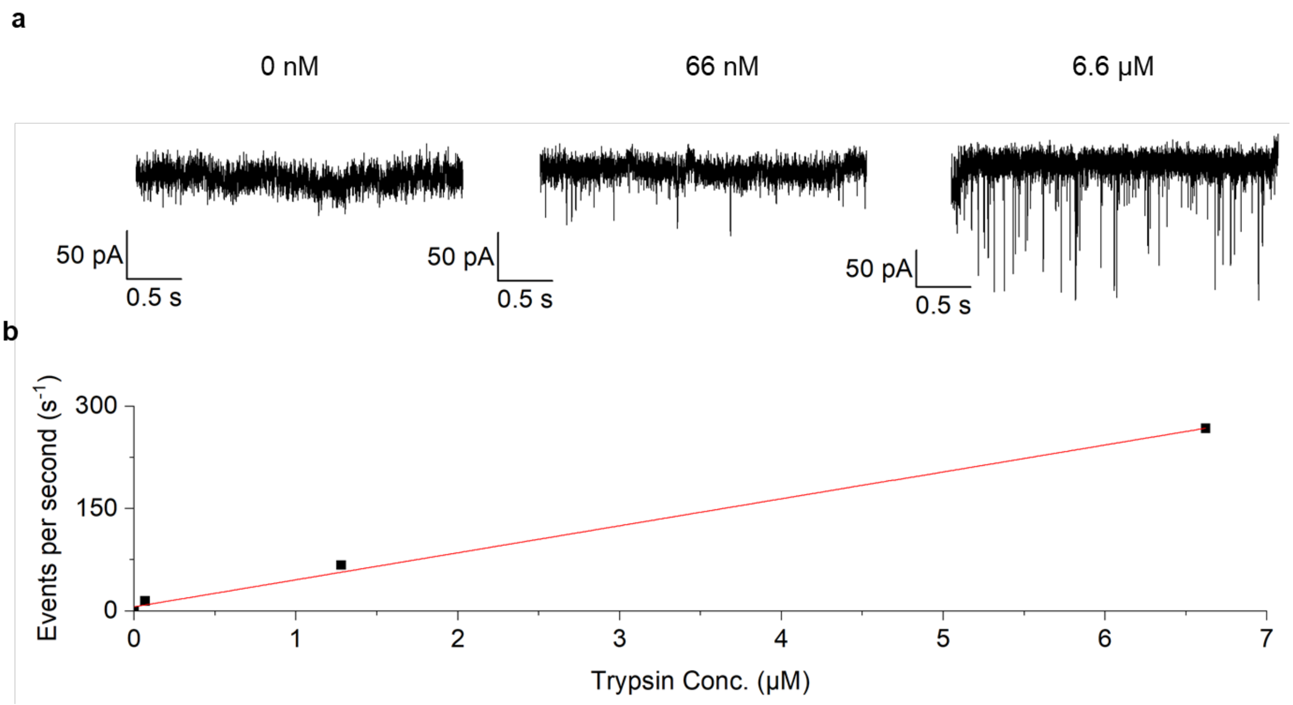

Supplementary Figure 33: Concentration-dependent trypsin translocation through LGC-N at 70 mV. (**a**) Example traces showing increasing concentrations of trypsin from 0 nM to 6.6 µM. (**b**) Graph showing the relationship between trypsin concentration and translocation events per second seen. Source data are provided as a Source Data file.

Analysis of the voltage-dependent translocation of trypsin through LGC-N

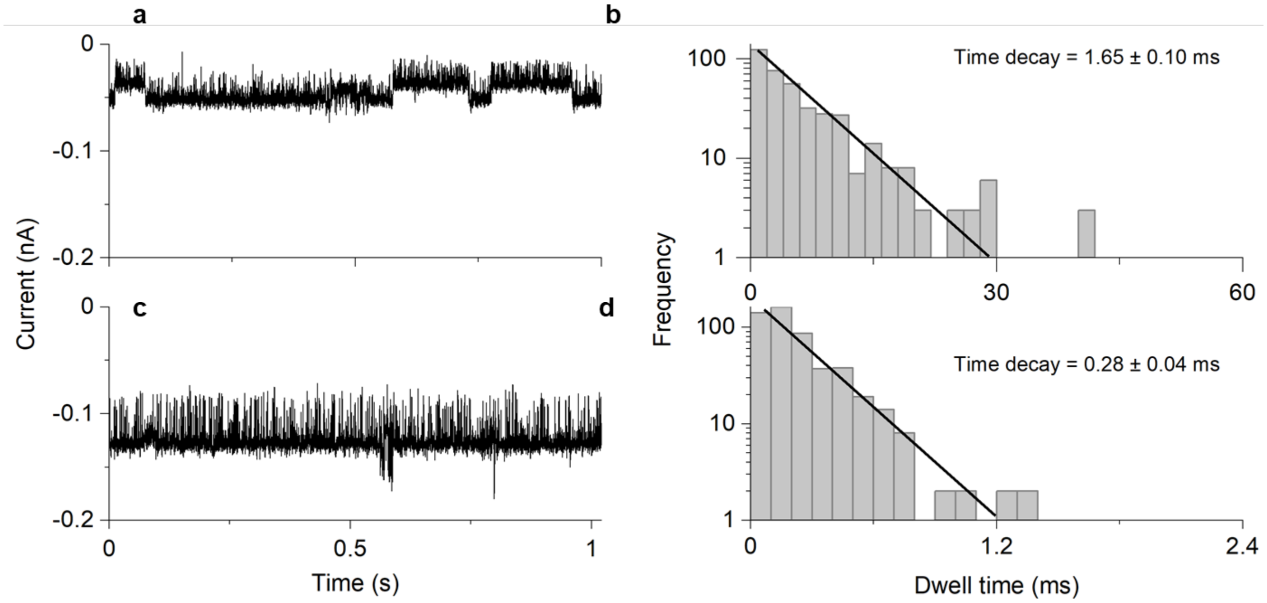

Supplementary Figure 34: Voltage-dependent trypsin translocation through LGC-N at (**a**-**b**) -20 mV and (**c**-**d**) -50 mV. (**a**+**c**) Example traces and (**b**+**d**) frequency histograms reveal that lower voltages result in a longer dwell time as shown by the time decay, calculated from the single-exponential fit to the dwell time frequency histograms. Source data are provided as a Source Data file.

pH dependence of GFP translocation through LGC-N

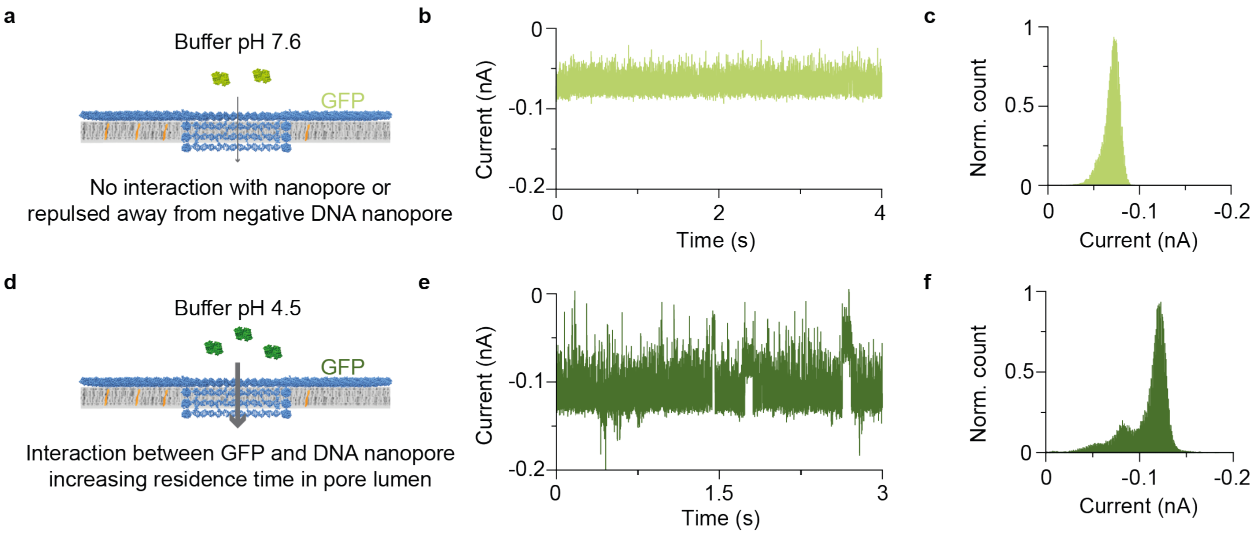

Supplementary Figure 35: (**a**) GFP translocation is not seen with normal electrophysiological buffers of pH 7.6 due to net negative charge of the GFP in these conditions. (**b**) LGC-N example trace after addition of GFP at pH 7.6. (**c**) Current frequency histogram showing a single peak after addition of GFP to LGC-N at pH 7.6. (**d**) At a buffer pH below the pI of GFP translocation events can now be resolved. (**e**) LGC-N example trace after addition of GFP at pH 4.5, showing an increase in translocation events. (**f**) Current frequency histogram showing multiple peaks, indicative of the different current levels caused by translocating GFP. Source data are provided as a Source Data file.

Analysis of GFP translocation through LGC-N at pH 4.5

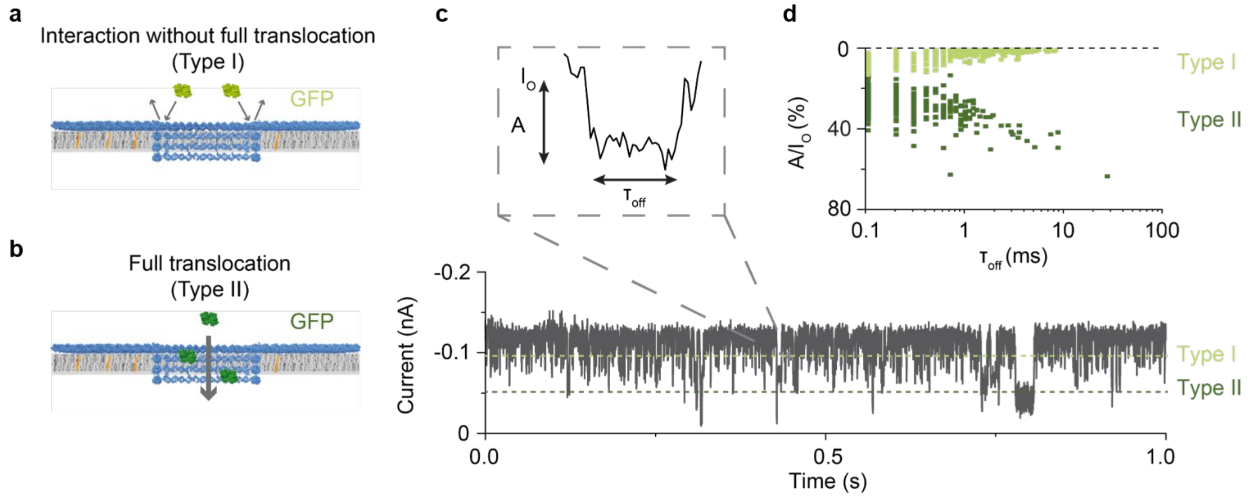

Supplementary Figure 36: (**a**) GFP translocation clusters into type I and type II events. Type I events are predicted to be GFP interaction with the nanopore lumen, without full translocation. (**b**) Type II events are predicted to be fully translocating GFP. (**c**) Example trace showing translocation events after addition of GFP at pH 4.5. Inset shows a single isolated translocation event with respect to amplitude (A), open current (I_O) and dwell time (τ_{off}). (**d**) Scatter plot representing τ_{off} and A, each point in the diagram represents an individual encounter event of protein with the DNA nanopore. Points cluster into type I (light green) and type II (dark green) events. Source data are provided as a Source Data file.

Size comparison of the ions and dye used to the oxDNA simulated LGC-C

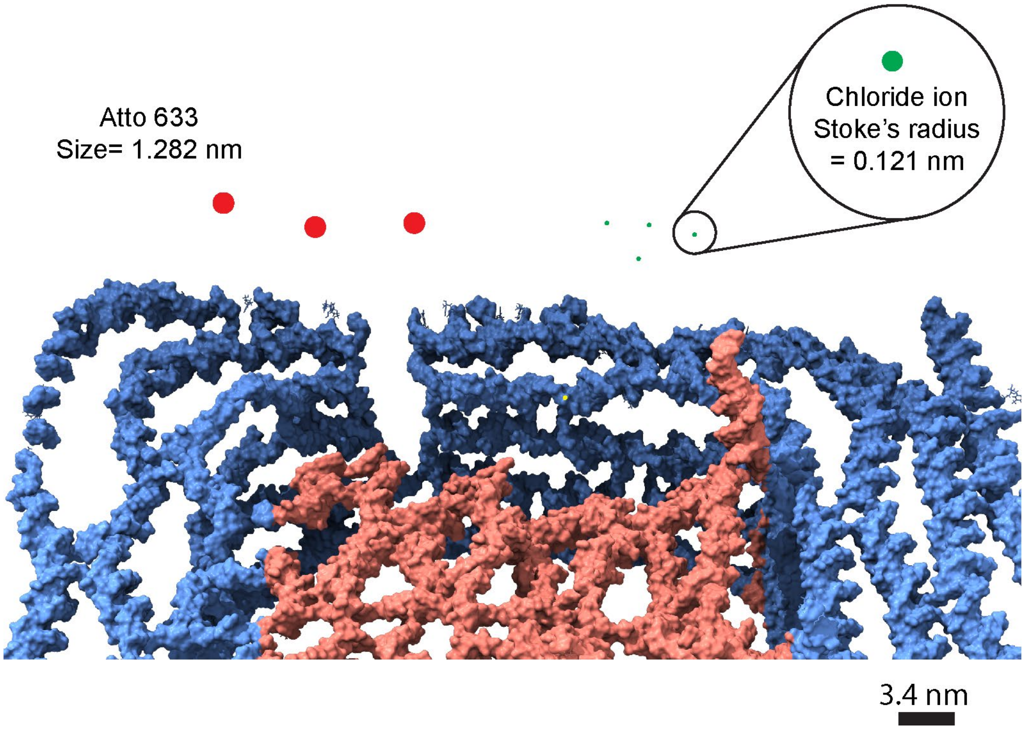

Supplementary Figure 37: Comparing the sizes of Chloride ion (green) and Atto 633 dye (red) used in electrophysiological measurements and dye influx confocal assay respectively to the close up of LGC-C (plate-blue, lid-pink). The scale bar is 3.4 nm or the length of one turn of B-form duplex DNA. Dye transport studies (Figure 3b) show that the closed lid nanopore is not permeable to the larger dyes (Atto 633 diameter = 1.3 nm), suggesting the leak of ions only (chloride ion Stoke’s radius = 0.1 nm).

Supplementary References

1. Diederichs, T., Pugh, G., Dorey, A., Xing, Y., Burns, J. R., Hung Nguyen, Q., Tornow, M., Tampé, R. & Howorka, S. Synthetic protein-conductive membrane nanopores built with DNA. *Nat. Commun.* **10**, 5018, (2019).
2. Thomsen, R. P., Malle, M. G., Okholm, A. H., Krishnan, S., Bohr, S. S. R., Sørensen, R. S., Ries, O., Vogel, S., Simmel, F. C., Hatzakis, N. S. & Kjems, J. A large size-selective DNA nanopore with sensing applications. *Nat. Commun.* **10**, 5655, (2019).
3. Krishnan, S., Ziegler, D., Arnaut, V., Martin, T. G., Kapsner, K., Henneberg, K., Bausch, A. R., Dietz, H. & Simmel, F. C. Molecular transport through large-diameter DNA nanopores. *Nat Commun* **7**, 12787, (2016).
4. Müller, C. B., Loman, A., Pacheco, V., Koberling, F., Willbold, D., Richtering, W. & Enderlein, J. Precise measurement of diffusion by multi-color dual-focus fluorescence correlation spectroscopy. *EPL (Europhysics Letters)* **83**, (2008).
5. Dertinger, T., Pacheco, V., von der Hocht, I., Hartmann, R., Gregor, I. & Enderlein, J. Two-focus fluorescence correlation spectroscopy: A new tool for accurate and absolute diffusion measurements. *ChemPhysChem* **8**, 433-443, (2007).
6. Liu, M., Jiang, S., Loza, O., Fahmi, N. E., Šulc, P. & Stephanopoulos, N. Rapid photoactuation of a DNA nanostructure using an internal photocaged trigger strand. *Angew. Chem. Int. Ed.* **57**, 9341-9345, (2018).
