# Supplementary material for: A reversibly gated protein-transporting membrane channel made of DNA
Source: Nat Commun. 2022 Apr 28;13:2271. doi: 10.1038/s41467-022-28522-2 (PMC9051096; doi:10.1038/s41467-022-28522-2)
Supplement: Supplementary file 3 — Description of Additional Supplementary Files [file 41467_2022_28522_MOESM3_ESM.docx]

**List of Additional Supplementary files provided:**

1. **File Name: Supplementary Data 1**Description: This file contains the sequences of all the strands used in the experiments carried out in the manuscript. It also has details on how to anneal the structures.
2. **File name: Supplementary video 1**

Description: This is a video file showing the OxDNA simulated structures of LGC-N (left), LGC-C (middle), and LGC-O (right) in solution.

1. **File name: Supplementary video 2**

Description: This is a video file showing confocal imaging of GUVs and Atto 633 with LGC-N +chol over 3 hrs. (red – Atto 633 dye, green – LGC)

1. **File name: Supplementary video 3**

Description: This is a video file showing confocal imaging of GUVs and Atto 633 with LGC-N -chol over 3 hrs. (red – Atto 633 dye, green – LGC)

1. **File name: Supplementary video 4**

Description: This is a video file showing confocal imaging of GUVs and Atto 633 with LGC-C +chol over 3 hrs. (red – Atto 633 dye, green – LGC)

1. **File name: Supplementary video 5**

Description: This is a video file showing confocal imaging of GUVs and Atto 633 with LGC-O +chol over 3 hrs. (red – Atto 633 dye, green – LGC)

1. **File name: Supplementary video 6**

Description: This is a video file showing confocal imaging of GUVs and Atto 633 with dynamically closed LGC-O +chol over 3 hrs. (red – Atto 633 dye, green – LGC)

1. **File name: Supplementary video 7**

Description: This is a video file showing confocal imaging of GUVs and Atto 633 with LGC-C -chol over 3 hrs. (red – Atto 633 dye, green – LGC)

1. **File name: Supplementary video 8**

Description: This is a video file showing confocal imaging of GUVs and GFP with LGC-N +chol over 3 hrs. (green- GFP, red- LGC)

1. **File name: Supplementary video 9**

Description: This is a video file showing confocal imaging of GUVs and GFP with LGC-C +chol over 3 hrs. (green- GFP, red- LGC)

1. **File name: Supplementary video 10**

Description: This is a video file showing confocal imaging of GUVs and GFP with LGC-O +chol over 3 hrs. (green- GFP, red- LGC)

1. **File name: Supplementary video 11**

Description: This is a video file showing confocal imaging of GUVs and GFP with dynamically closed LGC-O +chol over 3 hrs. (green- GFP, red- LGC)

1. **File name: Supplementary video 12:**

Description: This is a video file showing confocal imaging of GUVs and GFP with LGC-C -chol over 3 hrs. (green- GFP, red- LGC)
