# Supplementary material for: A reversibly gated protein-transporting membrane channel made of DNA
Source: Nat Commun. 2022 Apr 28;13:2271. doi: 10.1038/s41467-022-28522-2 (PMC9051096; doi:10.1038/s41467-022-28522-2)
Supplement: Supplementary file 17 — Reporting Summary [file 41467_2022_28522_MOESM17_ESM.pdf]

nature research

Corresponding author(s): Hao Yan, Stefan Howorka

Last updated by author(s): January 12, 2022

Reporting Summary

Nature Research wishes to improve the reproducibility of the work that we publish. This form provides structure for consistency and transparency in reporting. For further information on Nature Research policies, see our Editorial Policies and the Editorial Policy Checklist.

Statistics

For all statistical analyses, confirm that the following items are present in the figure legend, table legend, main text, or Methods section.

n/a	Confirmed	
☐	☒	The exact sample size (n) for each experimental group/condition, given as a discrete number and unit of measurement
☐	☒	A statement on whether measurements were taken from distinct samples or whether the same sample was measured repeatedly
☒	☐	The statistical test(s) used AND whether they are one- or two-sided<br>*Only common tests should be described solely by name; describe more complex techniques in the Methods section.*
☒	☐	A description of all covariates tested
☒	☐	A description of any assumptions or corrections, such as tests of normality and adjustment for multiple comparisons
☐	☒	A full description of the statistical parameters including central tendency (e.g. means) or other basic estimates (e.g. regression coefficient) AND variation (e.g. standard deviation) or associated estimates of uncertainty (e.g. confidence intervals)
☒	☐	For null hypothesis testing, the test statistic (e.g. F, t, r) with confidence intervals, effect sizes, degrees of freedom and P value noted<br>*Give P values as exact values whenever suitable.*
☒	☐	For Bayesian analysis, information on the choice of priors and Markov chain Monte Carlo settings
☒	☐	For hierarchical and complex designs, identification of the appropriate level for tests and full reporting of outcomes
☒	☐	Estimates of effect sizes (e.g. Cohen's d, Pearson's r), indicating how they were calculated

Our web collection on statistics for biologists contains articles on many of the points above.

Software and code

Policy information about availability of computer code

Data collection: Nikon Elements (NIS ElementsAR ver. 4.6.0.) was used to acquire images. Current recordings were acquired using Element Data Recorder software (version 3.8.18)(Element, s.r.l, Italy)

Data analysis: Origin Pro 2019 was used to analyze graphical data, Nikon Elements (NIS ElementsAR ver. 4.6.0.) was used to analyze CLSM images.

For manuscripts utilizing custom algorithms or software that are central to the research but not yet described in published literature, software must be made available to editors and reviewers. We strongly encourage code deposition in a community repository (e.g. GitHub). See the Nature Research guidelines for submitting code & software for further information.

Data

Policy information about availability of data

All manuscripts must include a data availability statement. This statement should provide the following information, where applicable:

- Accession codes, unique identifiers, or web links for publicly available datasets
- A list of figures that have associated raw data
- A description of any restrictions on data availability

All raw and processed data supporting the findings of this study are available within the paper, supplementary information files and the source data file.

Field-specific reporting

Life sciences study design

All studies must disclose on these points even when the disclosure is negative.

Sample size	No sample size calculation was performed. The sample size was chosen according to sample size used by previously published reports of similar experiments (N=3) (Krishnan et. al. 2016, Nat. Comm.)
Data exclusions	In the influx % calculation, the GUVs that were out of focus in the field of view under study were excluded from the analysis.
Replication	All replication attempts were successful and showed the influx trend expected. CLSM experiments were replicated 3 times each.
Randomization	No participants were involved in the study therefore randomization is not applicable
Blinding	Blinding is not carried out as the prepared samples were analyzed by the same person.

Reporting for specific materials, systems and methods

We require information from authors about some types of materials, experimental systems and methods used in many studies. Here, indicate whether each material, system or method listed is relevant to your study. If you are not sure if a list item applies to your research, read the appropriate section before selecting a response.

Materials & experimental systems

n/a	Involved in the study
☒	☐ Antibodies
☒	☐ Eukaryotic cell lines
☒	☐ Palaeontology and archaeology
☒	☐ Animals and other organisms
☒	☐ Human research participants
☒	☐ Clinical data
☒	☐ Dual use research of concern

Methods

n/a	Involved in the study
☒	☐ ChIP-seq
☒	☐ Flow cytometry
☒	☐ MRI-based neuroimaging
